# Idiosyncratic invasion trajectories of human bacterial pathogens facing temperature disturbances in soil microbial communities

**DOI:** 10.1038/s41598-024-63284-5

**Published:** 2024-05-29

**Authors:** Ascensio-Schultz Eliette, Barbier Elodie, Mounier Arnaud, Raynaud Tiffany, Spor Aymé, Piveteau Pascal

**Affiliations:** 1 Université de Bourgogne, University Bourgogne Franche-Comté, INRAE, Institut Agro, Agroécologie, 21000 Dijon, France; 2 INRAE, UR OPAALE, Rennes, France

**Keywords:** Microbial ecology, Invasion biology, Soil, *Listeria monocytogenes*, *Klebsiella pneumoniae*, Soil microbiology, Microbial ecology

## Abstract

Current knowledge about effects of disturbance on the fate of invaders in complex microbial ecosystems is still in its infancy. In order to investigate this issue, we compared the fate of *Klebsiella pneumoniae* (Kp) and *Listeria monocytogenes* (Lm) in soil microcosms. We then used environmental disturbances (freeze–thaw or heat cycles) to compare the fate of both invaders and manipulate soil microbial diversity. Population dynamics of the two pathogens was assessed over 50 days of invasion while microbial diversity was measured at times 0, 20 and 40 days. The outcome of invasion was strain-dependent and the response of the two invaders to disturbance differed. Resistance to Kp invasion was higher under the conditions where resident microbial diversity was the highest while a significant drop of diversity was linked to a higher persistence. In contrast, Lm faced stronger resistance to invasion in heat-treated microcosms where diversity was the lowest. Our results show that diversity is not a universal proxy of resistance to microbial invasion, indicating the need to properly assess other intrinsic properties of the invader, such as its metabolic repertoire, or the array of interactions between the invader and resident communities.

## Introduction

Biological invasion, the process by which invading species settle in an ecosystem, is a dynamic field of research in Ecology. Much attention has been paid on biological invasion of terrestrial ecosystems^1–3^ because it is one of the critical processes that shape ecosystems’ biomes. Microbial invasion refers to the process in which introduction of invading microorganisms results in establishment, growth and spread of the invasive species eventually leading to impacts on the ecosystem^4,5^. Indeed, invasive microorganisms can alter resident community structure and possibly functioning in a soil-dependent and invader-dependent manner^6–9^.

Upon introduction of invading microorganisms into soil, establishment depends on edaphic characteristics. Abiotic factors such as pH^10^ and soil texture^11,12^ influence the success of microbial invasion. Interestingly, invasive success is dependent on the availability of nutrients, on resource stoichiometry and on the ability to utilise the available nutrients, connecting niche breadth to invasion success^13–15^. This relationship depends on the native microbial diversity and community structure which are key components of the soil resilience to microbial invasion^10,16–19^. A large array of interspecific interactions could underlie this diversity-invasion resistance effect spanning from antibiosis^20–23^ to exploitation competition^14,15^.

Conversely, introduction of invading bacteria in soil affects a native community’s diversity, as well as its structure and niche breadth^7–9^, even if invading bacteria fail to settle^24^. Because microbial diversity is directly affected by habitat disturbances^25–27^, the diversity-invasion resistance relationship in soil can be modified following disturbance. Indeed, microbial community members react differently to disturbance^27^ and disturbance history leads to legacy effects that affect soil microbiome functioning^25,26, 28^. For example, we previously reported that heat disturbances (+ 42 °C) reduced Lm invader survival but cold disturbance (− 20 °C) increase Lm invader survival relative to ambient conditions (+ 20 °C throughout the incubation) and the invader persisted until the end of the experiment^12^. Heat disturbances resulted in a significant drop of microbial diversity but high microbial diversity was still recorded after mild freezing treatments. Intriguingly, these invasion results did not fully agree with the assumption that survival of the invader is expected to be facilitated when resident diversity is low and it suggests that disturbance-induced shifts in resident community membership rather than diversity was the main driver of the soil resistance to invasion by *L. monocytogenes*. In order to confirm the generality of this effect, we designed invasion experiments with an invading species phylogenetically distant from *Listeria monocytogenes*. We selected the ubiquitous species *Klebsiella pneumoniae*, a species that includes emerging pathogens causing new human health burdens^29,30^. *K. pneumoniae* environmental reservoirs include food products, water, and soil^31–36^. There is concern that virulent Kp strains could be found in environmental reservoirs^37–40^ but the contribution of environmental reservoirs to the occurrence of disease is still under debate^32,41, 42^. We used temperature disturbances to promote time-dependent shifts in stochastic and deterministic balances of soil microbial communities. We hypothesised that the patterns of invasion by *Klebsiella pneumoniae* during temperature disturbances were similar to what was previously observed for *Listeria monocytogenes*: survival in cold disturbed habitats higher than in heat disturbed habitats (hypothesis 1). We further hypothesised that the consequences of invasion on resident microbial communities were similar for both invaders (hypothesis 2).

## Results

### Dynamics of Kp invasion during disturbances differ from those of Lm

**Figure 1:**
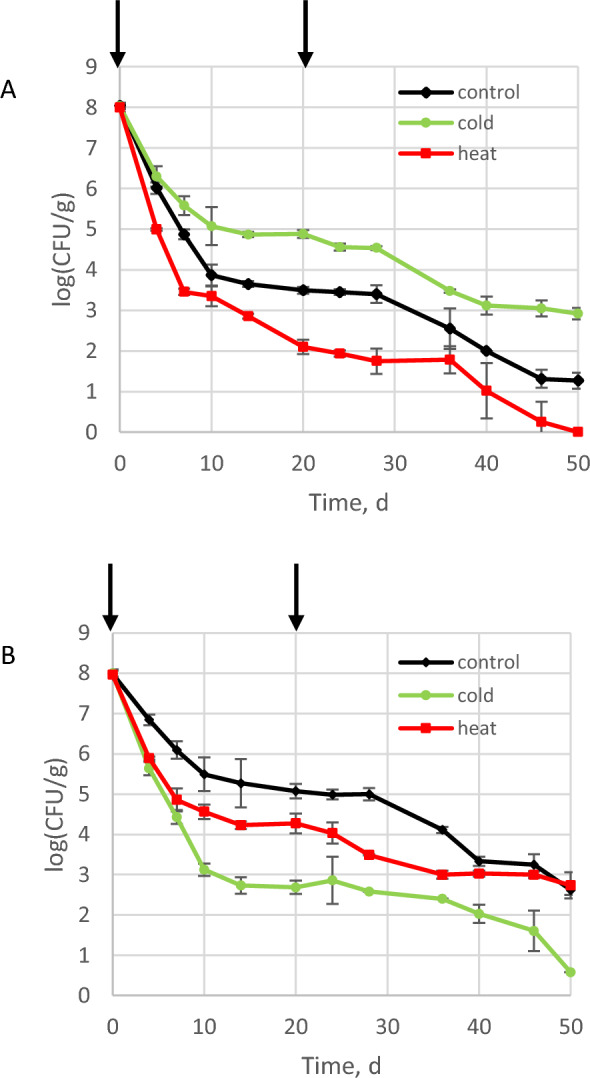
Fate of the population of *L. monocytogenes* L9 (**A**) and *K. pneumoniae* MGH 78,578 (**B**) during invasion of soil microcosms. Arrows indicate the time when disturbance was applied.

To address our working hypothesis that Kp would follow invasion dynamics similar to Lm i.e. better survival in cold than in heat treatment, experiments were run in soil microcosms. When inoculated into soil microcosms in presence of the resident microbial community, whatever the treatment, the populations of the invader decreased during the time of incubation (Fig. 1).

In order to confirm the reproducibility of our previous results with Lm^12^ and the reliability of our experimental design, Lm invasion trials were run. The current data confirmed the results collected previously^12^. A three-way interaction between disturbance, invasion treatment and time was significant (*p* = 0.001) and differences between disturbance treatments were significant (*p* = 0.001). Significantly higher populations were numerated in cold-disturbed microcosms in comparison to the other two treatments (Fig. 1A, Supplementary Figs. S1A,S7A). For example, after 2 weeks of incubation the population of Lm in cold-disturbed microcosm was 7.4 10^4^ ± 1.1 10^4^ CFU/g. The Lm population was significantly lower in the heat-disturbed microcosms (7.2 10^2^ ± 9.6 10^1^ CFU/g) compared to the cold-disturbed and control microcosms (4.5 10^3^ ± 6.9 10^2^ CFU/g) and this disturbance led to the absence of detection of Lm after 46 days of incubation (Fig. 1A). Similar results were found during co-invasion by Kp and Lm (Supplementary Fig. S1).

As for Lm, during Kp invasion of soil microcosms, there was a statistically significant three-way interaction between disturbance treatment, invasion treatment and time (*p* = 0.001). Interaction between treatment and time was significant (*p* = 0.001). A statistically significant effect of the disturbance was found (*p* < 0.0001) but differences between invasion treatment were not significant (*p* = 0.258). Unlike what was recorded during Lm invasion trial, Kp populations were significantly lower in all disturbed microcosms than in undisturbed control microcosms (1.1 10^6^ ± 1.2 10^6^ CFU/g at day 14) (Fig. 1B, Supplementary Figs. S1B,S8A). Moreover, in contrast with Lm, Kp population in cold-treated microcosms (3.6 10^3^ ± 1.5 10^3^ CFU/g at day 14) was significantly lower than in heat-treated microcosms (5.5 10^4^ ± 1.3 10^4^ CFU/g).

**Figure 2:**
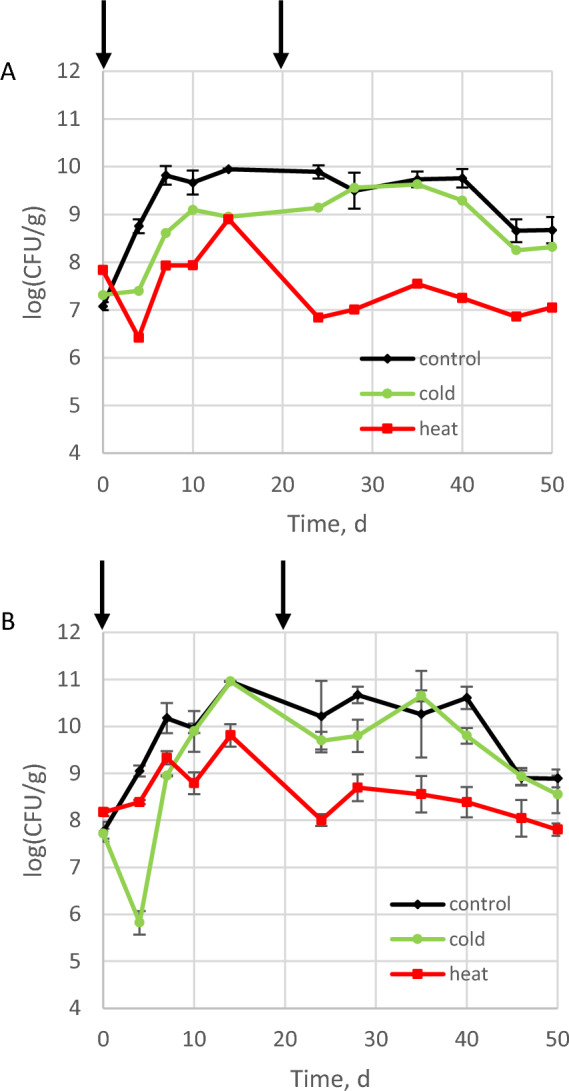
Fate of the population of *L. monocytogenes* L9 (**A**) and *K. pneumoniae* MGH 78,578 (**B**) during invasion of sterilised soil microcosms. Arrows indicate the time when disturbance was applied.

Sterilised soil microcosms were used to assess the direct impact of temperature disturbances on the invader population. Growth of Lm and Kp in sterilised soil incubated at constant temperature suggested that both strains were able to utilise resources available in soil (Fig. 2A,B). Metabolic profiles of Lm and Kp were significantly different (Supplementary Fig. S6). Both invading strains displayed significant differences (*p* < 0.05) in their ability to utilise substrate categories amine, amino acids, carbohydrates, carboxylic acid, but similar degradation of polymer was measured (*p* > 0.05).

The data collected during Lm invasion of sterilised soil confirmed our previous results ^12^. Indeed, in sterile soil microcosms inoculated with Lm, the three-way interaction between disturbance treatment, invasion treatment and time was significant (*p* = 0.001). Significant differences were found between disturbances (0.0001). Either cold or heat treatments at the start of the incubation resulted in lower populations of Lm (day 10: 1.3 10^9^ ± 3.1 10^8^ CFU/g in cold-disturbed and 9.4 10^7^ ± 4.5 10^7^ CFU/g in heat-disturbed) in comparison to the undisturbed controls (5.2 10^9^ ± 2.3 10^9^ CFU/g) and differences remained significant during the first ten days of incubation (Fig. 2A). Lm populations were significantly lower in heat-disturbed than in cold-disturbed microcosms during this period. The second round of disturbances resulted in a significant decrease of the population of Lm in heat-disturbed microcosms (day 28: 1.1 10^8^ ± 3.8 10^6^ CFU/g) in comparison with cold-disturbed microcosms (day 28: 3.6 10^9^ ± 4.7 10^8^ CFU/g) and control (day 28: 4.1 10^9^ ± 2.9 10^9^ CFU/g) (Figs. 2A, S7B). Moreover, differences between cold-disturbed and undisturbed control microcosms were no longer significant until the end of the experiment.

Similarly, considering Kp populations, a three-way interaction between disturbance treatment, invasion treatment and time was observed (*p* = 0.001). Differences between disturbance treatments were significant (*p* = 0.0001) unlike the invasion treatment (*p* = 0.320). During the first ten days of incubation Kp populations were significantly lower in disturbed microcosms than in control microcosms (day 4: 1.1 10^9^ ± 3.1 10^8^ CFU/g) (Figs. 2B, S8B). Unlike what was observed with Lm, the impact of freezing was harsher (day 4: 7.4 10^5^ ± 3.9 10^5^ CFU/g) than heat (day 4: 2.5 10^8^ ± 2.6 10^7^ CFU/g) but Kp populations recovered steadily from freezing and after two weeks on incubation, differences between control and cold treatment were not significant. On the opposite lower Kp population was recorded in heat microcosms (Figs. 2B, S8B).

When the two invading strains were inoculated together in sterile soil, the effect of the disturbances on both invading strains followed similar trends (Supplementary Fig. S2).

### Resident communities’ response to disturbances and invader species invasion

**Figure 3:**
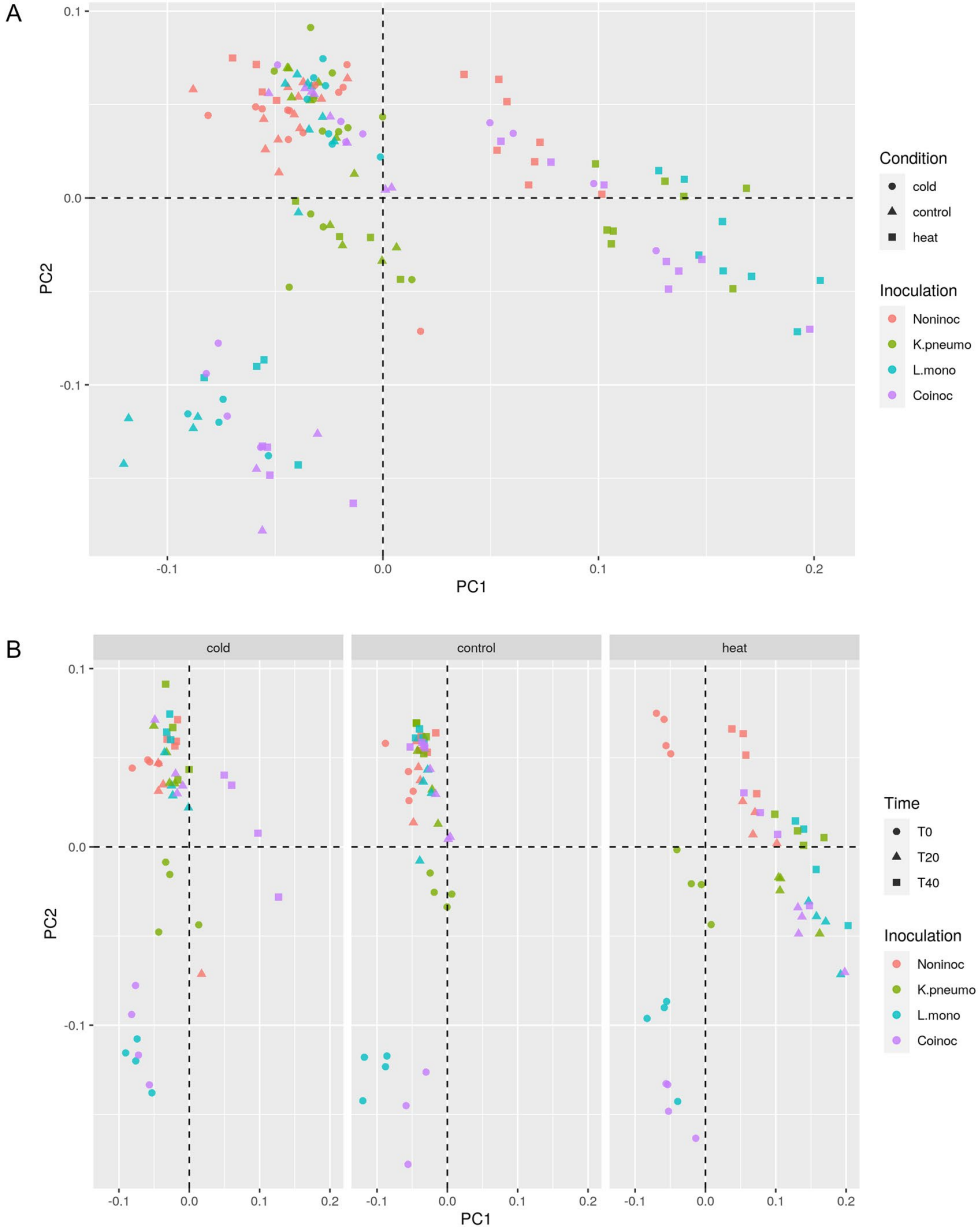
(**A**) Effect of disturbance treatments on bacterial community composition in soil microcosms facing ongoing invasion. Differences in community composition were estimated using the weighted UniFrac distance. This figure represents a PcoA of the weighted UniFrac distance matrix. Circles, triangles and squares correspond respectively to cold, control and heat treatments. Red: no invasion; green: *K. pneumoniae* MGH 78,578 invasion; blue: *L. monocytogenes* L9; purple co-invasion of *K. pneumoniae* MGH 78,578 and *L. monocytogenes* L9. (**B**) Effect of invasion conditions on bacterial community composition in soil microcosms during incubation. Differences in community composition were estimated using the weighted UniFrac distance. This figure represents a PcoA of the weighted UniFrac distance matrix. Circles, triangles and squares correspond respectively to T0, T20 and T40. Red: no invasion; green: *K. pneumoniae* MGH 78,578 invasion; blue: *L. monocytogenes* L9; purple co-invasion of *K. pneumoniae* MGH 78,578 and *L. monocytogenes* L9.

In microcosms without inoculation, variations of the overall diversity of resident microbiota were limited in control and cold-disturbed microcosms, but heat disturbance resulted in a significant decrease of diversity (Supplementary Figs. S3A-C,S4A-C,S5A-C) and a significant shift in bacterial community structure (PERMANOVA, Disturbance effect R^2^ = 28.8, *p* < 0.001; Fig. 3A,B).

**Figure 4:**
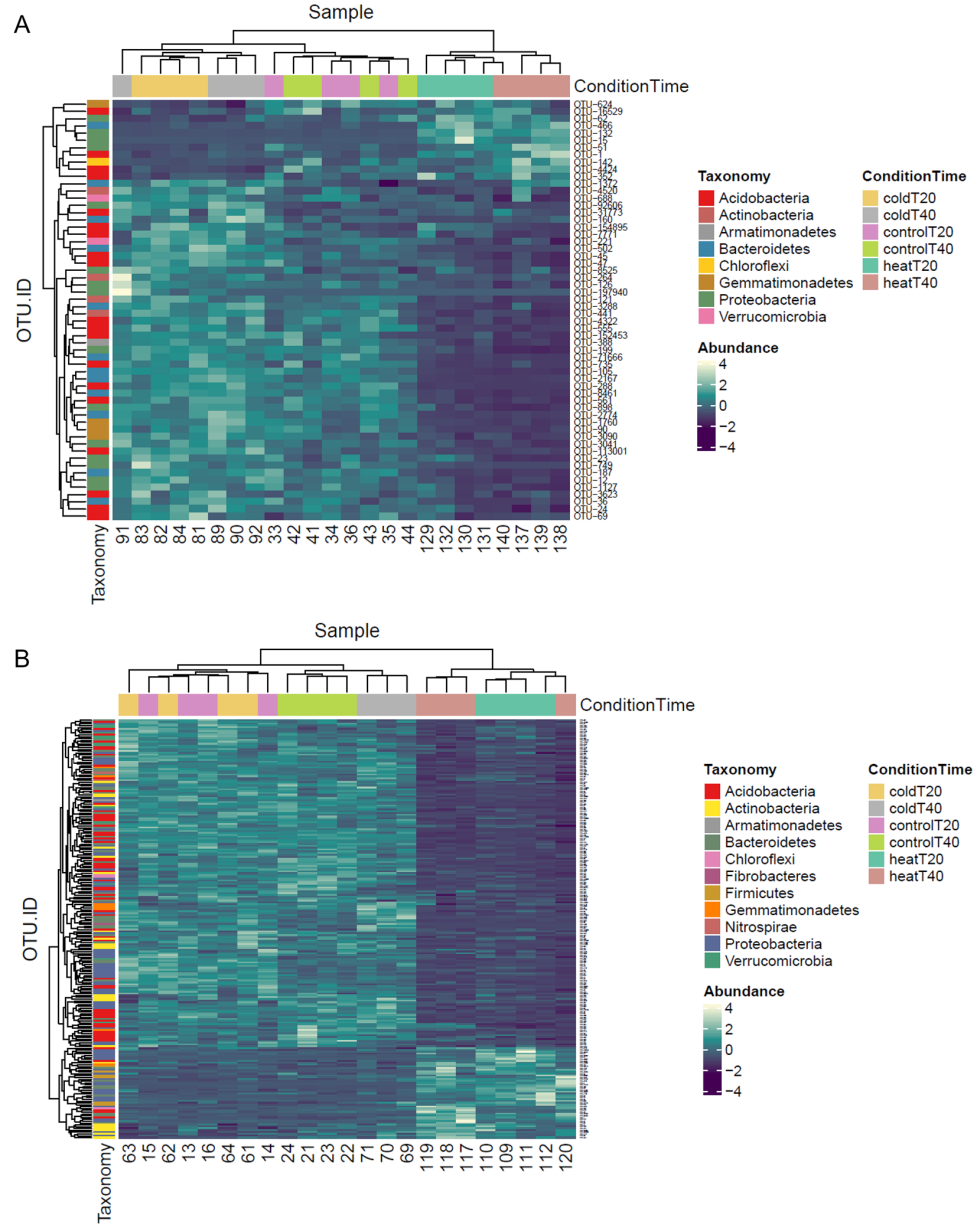
Relative abundance of OTUs significantly associated with the invasion success of the two invading bacteria across the different disturbance treatments and times of incubation. (**A**) *K. pneumoniae* MGH 78,578 (**B**) *L. monocytogenes* L9.

**Figure 5:**
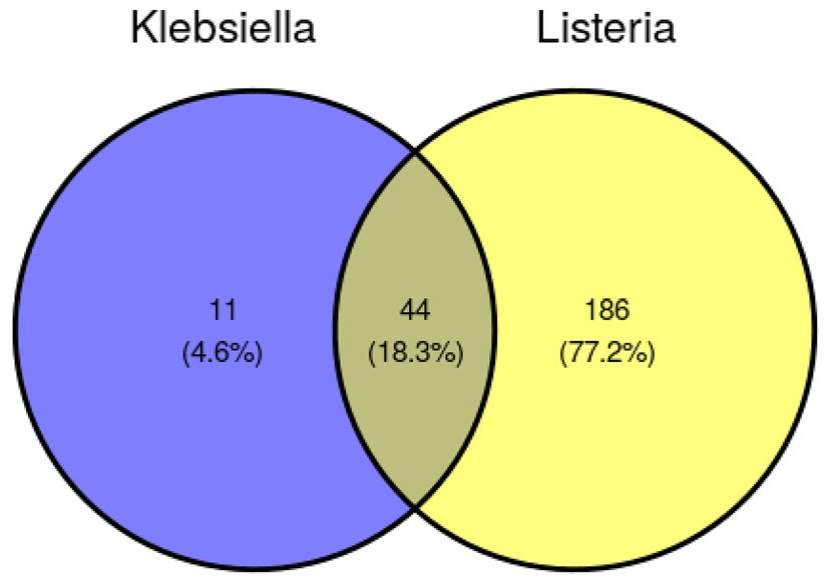
Venn diagram of the number of OTUs significantly different from non-inoculated treatments.

Conditions of invasion further affected microbiota. Invasion by Kp resulted in significantly lower variations of the relative abundance of OTUs than invasion by Lm (Figs. 4A,B, 5). In control microcosms, alpha-diversity indices were not impacted by the invasion treatment at T20 and T40 (Tukey HSD tests, *p* > 0.05; Supplementary Fig. S3A-C,S4A-C,S5A-C) and overall, the invasion treatment had no strong impact on bacterial community structure (PAIRWISE PERMANOVA *p* > 0.05; Fig. 3B). In cold treatments, co-invasion with Lm and Kp was the only condition to generate significant differences of alpha-diversity (Tukey HSD tests, *p* < 0.05; Supplementary Figs. S3C,S4C,S5C) and of bacterial community structure (PAIRWISE PERMANOVA *p* < 0.05; Fig. 3B), but only at T40. In heat treatments, the invasion treatment had a slight but significant effect on the number of observed species after 40 days, that was increased in the co-invasion treatment compared to the other ones (Supplementary Fig. S3C). The invasion treatment had, however, a strong impact on bacterial community structure at T20 and T40, with all inoculation treatments displaying significant differences in terms of composition compared to the non-inoculated one (PAIRWISE PERMANOVA *p* < 0.05; Fig. 3B). It should be pointed out here that overall, the effects on alpha-diversity indices are relatively small and become insignificant after multiple comparison corrections, probably due to our small sample size.

### Correlation between soil microbial community composition and the success of invasion

Clustering of the OTUs table according to the fate of Kp (Fig. 4A) and Lm (Fig. 4B) clearly showed that statistically significant variations of OTUs correlated with the fate of the invasive species. Under the least permissive conditions for Kp (cold-disturbed microcosms), 45 OTUs with significantly higher abundance were identified, while a limited number of OTUs (10) with high abundance was observed in the more permissive heat-disturbed condition (Fig. 4B, Supplementary Table S2). Among the 45 identified OTUs, 4 belong to the phylum Actinobacteria.

The strongest decrease of Lm population observed in the heat treatment correlated with a group of 50 OTUs with higher abundance than recorded in the more permissive cold treatment (Fig. 4A, heat, Supplementary Table S1). Fourteen percent of these 50 OTUs belong to the phylum Actinobacteria. Conversely, a large group of 180 OTUs with high abundance was identified under the conditions permissive for Lm (Fig. 4A, cold, Supplementary Table S1).

## Discussion

One of microbial ecology’s major challenges is to establish conceptual frameworks in order to comprehend fully microbial communities^43^. In the field of invasion biology, microorganisms have become model organisms to investigate the drivers of the resistance of resident communities to invading organisms^4,5, 44, 45^.

In the present study, we assessed the response of an invading pathogen, *Klebsiella pneumoniae*, to a disturbed soil habitat and compared our results to results collected from *Listeria monocytogenes* invasion. We set up experiments based on temperature shifts and assessed their impacts on invader populations and on resident community diversity and composition. Disturbances considered here are representative of realistic environmental scenarios and led to results that are in line with previous reports. Indeed, conclusions on the fate of the invader Lm and the soil community’s response to invasion were similar to our former study^12^. However, in contradiction with our working hypothesis, Kp displayed different trends. In contrast to the positive effect of cold on Lm invasion, there was a strong negative effect of freezing on Kp invasion. Kp and Lm are mesophilic species. Their optimum temperature is 37 °C and their maximum growth temperature is around 42 °C^46–49^. One distinctive trait of Lm is its ability to grow at 4 °C and below^50–52^ unlike Kp which is unable to grow at temperatures lower than 8 °C^49,53^. Freezing has detrimental effects on Lm^54^ and Kp^55^ but as seen in sterilised soil, under our experimental conditions, Kp was more sensitive to the cold treatment than Lm.

In invasion assays in soil microcosms, discrete disturbances were used to promote time-dependent shifts in the stochastic/deterministic balance of processes that promote resistance to invasion. Heat treatment affected microbial diversity and community structure as seen in non-inoculated microcosms. This is consistent with previous reports showing that habitat disturbance affects microbial communities^25–27, 56^. In the present study, we confirmed our previous observation that Lm invasion increased the consequences of heat disturbance on microbial diversity and community composition and that Lm had limited impact during invasion of cold-treated and control microcosms^12^. Such effects of the invader during disturbance was not verified during Kp invasion. This suggests that Lm and Kp engaged in distinct strategies upon their arrival in soil. In strongly disturbed heat-treated microcosms, the large metabolic repertoire of Kp probably participated to its better survival than in less disturbed conditions. Increased resource availability after disturbance could be one of the mechanisms that underlay disturbance-mediated invasion^57–59^. From the invader perspective, the capacity to utilise resources can promote invasion success^14,60^. For example, in reconstituted freshwater microcosms composed of eight bacterial species, niche and fitness differences between invaders and residents were good predictors of the invasion success^61^. On top of the direct effect of freezing on Kp population, the potential increase in abundance of Kp antagonistic OTUs in those treatments could be another mechanism that might explain the lower survival of Kp in cold-treated microcosms during invasion. Interestingly, we showed that among the 45 identified OTUs whose abundance was increased in the cold-treated microcosms, 4 belong to the phylum Actinobacteria. Several genera from this phylum produce inhibitory compounds active against Kp^62–70^. No information is however available on a potential antagonistic effect against Kp of the other OTUs detected as significantly increased in abundance in the COLD-treated microcosms.

In the present study we confirmed that Lm invasion does not comply with the diversity-invasion resistance framework. Heat disturbance affected soil communities but resulted in increased resistance against Lm invasion while cold disturbance had limited impact on diversity but facilitated invasion by Lm. Overrepresentation of a limited number of Lm competitors in heat-disturbed habitats could explain resistance to invasion. Indeed, we detected 50 OTUs whose abundance was significantly increased in heat-treated microcosms where survival of Lm was significantly lower. Among those, fourteen percent belonged to the Actinobacteria phylum. Actinobacteria produce bioactive compounds, including inhibitory compounds that were reported to be active against Lm^71^. Twelve percent of the detected OTUs are Firmicutes, including two occurrences of the genus *Bacillus*. Some *Bacillus* strains are known to produce inhibitory compounds active against Lm^72–74^. Competition is a driving force of community assembly^21^ and several mechanisms can trigger competition^17,22, 23^. For example, soil-dwelling bacteria trigger interference competition against *Listeria monocytogenes*^73,75, 76^. Similarly, interference competition was observed between bacterial communities and Lm in composts^77^. Considering that interference competition triggered by Lm has been reported in complex microbiota^78^, exclusion of competitors in cold-disturbed habitats could explain the fate of Lm under these circumstances.

According to these results, one can assume that specific combinations of microbial community composition and abiotic characteristics define permissive conditions facilitating persistence of Kp and/or Lm at low numbers in native soils as documented for Lm detection in produce field^79–81^. However in natural settings, to the best of our knowledge little information is currently available on how long a strain of Lm may persist after invasion of soil.

Altogether, our results show that several routes of invasion of microbial ecosystems are likely: the invasion trajectory of Kp in disturbed soils is different from Lm’s one. This is certainly related to the two species’ intrinsic properties, such as a different response to temperature shifts or a different niche breadth, but also to the resident community properties (*i. e.* its diversity and composition), as well as its response to environmental disturbances. Future research with more invaders displaying contrasted niche breadths, and the evaluation of the wide range of specific interactions invaders might engage in with members of resident communities are required for a deeper understanding of microbial invasion biology that definitely requires more than a measurement of resident community’s diversity.

## Materials and methods

### Experimental design

Soil was used as model habitat for invasion in order to address the consequences of temperature disturbances on biological invasion (hypothesis 1) and on resident communities (hypothesis 2). *L. monocytogenes* and *K. pneumoniae* were selected as the two invading bacterial species. Habitat disturbances were simulated by discrete thermal fluctuations, either freeze–thaw (− 20 °C) or temperature increase to 42 °C. Invasion of the soil habitat was monitored for 50 days at 20 °C under controlled conditions, with or without thermal disturbance and under several invasion treatment (Lm alone, Kp alone, Lm and Kp co-invasion, no invasion). Inoculated control microcosms were incubated at the constant temperature of 20 °C. Non-inoculated microcosms were processed accordingly to assess the impact of disturbances on native microbial communities in the absence of invasion. Shifts of microbial diversity in the course of incubation of inoculated and non-inoculated microcosms were assessed through 16S rRNA gene amplicon sequencing.

Sterilised soil microcosms were included in the experimental design in order to decipher between the contribution of the biotic context and disturbances on the fate of the invading species.

### Invader bacterial strains and inoculum preparation

Kp and Lm were selected as invaders because of their reported occurrence in soil habitats and relevance for public health. *Klebsiella pneumoniae* MGH 78,578, from the Pasteur Institute, Paris, was used as one invading species. This strain of *K. pneumoniae* from clinical origin was isolated in 1994 from a sputum of an American patient with pneumonia in an intensive care unit. The second invader was *Listeria monocytogenes* L9, a rifampicin resistant derivative of the type strain *L. monocytogenes* EGD-e^82^. Frozen stock cultures were stored at − 80 °C. A first overnight culture of 10 mL of Trypton Soy Broth (TSB; Conda, Spain) was prepared from the stock culture of each strain and incubated 24 h at 20 °C. These cultures were then transferred in fresh TSB (1% v/v) and incubated 24 h at 20 °C. Grown cultures were centrifuged (10,000 g, 10 min, room temperature) and the pellets suspended in sterile distilled water.

### Soil microcosms

A clay loamy soil with a pH of 7.15 was collected at INRAE experimental farming unit [Époisses, France, (47° 30ʹ 22.1832ʺ N, 4° 10ʹ 26.4648ʺ E)] nearby the location of the lab. On the collection site, four areas 20 m apart from each other were located. In each area, five soil cores (0–20 cm) were sampled and pooled into a composite sample. Soil was stored at 4 °C and processed within 48 h. Soil was sieved to 5 mm. *L. monocytogenes* and *K. pneumoniae* were not detected in the composite sample. Aliquots of the soil were sterilized by γ-radiation (45 kGy; Ionisos, Dagneux, France). Sterilized and non-sterile soil microcosms were prepared. Sterile soils allow investigating the sole effect of temperature disturbances, while non-sterile conditions were used to explore the effect of soil community diversity and composition on the invasion success of *L. monocytogenes* and *K. pneumoniae* in the presence and absence of disturbances.

Soil microcosms were prepared in 500 ml glass flasks with 150 g of soil. At the beginning of the experiment (T0), microcosms were inoculated with either *L. monocytogenes* (10^8^ CFU/g dry soil), *K. pneumoniae* (10^8^ CFU/g dry soil) or a mixture of both in the adequate water volume to reach 60% of the water holding capacity (WHC). After inoculation, microcosms were closed with sterile lids and population dynamics were followed for 50 days. Two environmental disturbances were considered: a freeze–thaw disturbance (− 20 °C) referred to ‘Cold’ and a Heat disturbance (+ 42 °C) referred to ‘Heat’. The duration of each temperature treatment was twice 30 h separated by 24 h at 20 °C. These treatments result in changes in microbial community composition of soil^56^. Non-inoculated microcosms were prepared accordingly as controls for rDNA 16S diversity analysis. The disturbances were applied in sterilized and non-sterile soil microcosms at the start of the experiment (T0) and after 20 days of incubation (T20) in the following sequence: ColdT0/ColdT20 (cold), and HeatT0/HeatT20 (heat). Control undisturbed sterilized and non-sterile soil microcosms (control) were incubated at the constant temperature of 20 °C for the total duration of the experiment. The soil moisture was checked after disturbances and during incubation. The water content was adjusted if required. For all experimental conditions, four independent replicates were considered.

### Numeration of *L*. *monocytogenes* and *K*. *pneumoniae* population

In order to enumerate *L. monocytogenes* and *K. pneumoniae* populations throughout incubation, 1 g equivalent dry weight of each microcosm was added to 9 ml tryptone salt (TS) and 0.7 g glass beads (Sigma, France). Microorganisms were suspended by agitation with a vortex operated full speed for 2 min. The soil slurry was serially diluted and plated onto « RAPID’L.mono » plates (BIORAD, France) and homemade Simmons Citrate Agar enriched with inositol plates^31^ for enumeration of *L. monocytogenes* and *K. pneumoniae* respectively. When required, pour plating was used for enumeration in order to improve sensitivity. The limit of detection of the assay was 10 CFU/g dry soil.

### Soil DNA extraction and 16S rRNA gene MiSeq sequencing

For each microcosm, total DNA was extracted from 250 mg dry soil with Fast DNA spin kit (MP Bio, France) according to the manufacturer’s manual. DNA’s integrity was assessed after electrophoresis on 1% agarose gel. Total DNA concentration was quantified by fluorometry using a Quant-iT PicoGreen dsDNA Assay Kit (Invitrogen, Cergy-Pontoise, France) following the manufacturer’s instructions. A two-step PCR procedure was used for library preparation. First of all the V3–V4 hypervariable region of bacterial 16S rRNA gene was PCR amplified with primers Pro341F (5’-TCGTCGGCAGCGTCAGATGTGTATAAGAGACAGNNNNCCTACGGGNBGCASCAG-3’) and Pro805R (5’-GTCTCGTGGGCTCGGAGATGTGTATAAGAGACAGNNNNGACTACNVGGGTATCTAATCC-3’)^83^. Duplicate 15 µL amplification reactions were prepared with 7.5 µL Phusion High-Fidelity PCR Master Mix (Thermo Fischer Scientific), 0.25 mM of each primer, 250 ng T4 gp32 (MPBio) and 1 ng template DNA. After 3 min at 98 °C, amplification proceeded during 25 cycles of 98 °C for 30 s, 55 °C for 30 s, and 72 °C for 30 s, with a final extension at 72 °C for 10 min. PCR products were pooled before the second PCR reaction in which multiplexing index sequences were added. This second PCR reaction was done in duplicate. A unique multiplex primer pair combination was used for each sample. The 30 µL reaction mixture was composed of 15 µL Phusion High-Fidelity PCR Master Mix (Thermo Fischer Scientific), 250 ng T4 gp32 (MPBio), 1 mM of one forward and one reverse multiplex primer and 6 µL of first step PCR product. Thermal cycling conditions were as for the first reaction except that the reaction was stopped after eight cycles. PCR products were pooled, checked on 1% agarose gel, cleaned-up, and purified. Quantified products were normalized with SequalPrep Normalization plates kit (Thermo Fisher Scientific) and pooled. These 203 samples were sent for sequencing on MiSeq (Illumina, 2 bp × 250 bp, MiSeq reagent kit v2, 500 cycles). Sequences were processed with Illumina MiSeq Reporter software (version 2.5.1.3) for demultiplexing and trimming of adaptors and barcodes.

### Bioinformatics analysis of 16 rRNA gene and rDNA diversity

The sequence data were analysed using an in-house developed Jupyter Notebooks pipeline^84^ piping together different bioinformatics tools. Briefly, R1 and R2 sequences were assembled using PEAR^85^ with default settings. Further quality checks were conducted using the QIIME pipeline^86^ and short sequences were removed (< 400 bp). Reference based and de novo chimera detection, as well as clustering in OTUs were performed using VSEARCH^87^ and the adequate reference databases (SILVA v132 representative set of sequences). The identity thresholds were set at 94% for 16S rRNA gene data based on replicate sequencing of a bacterial mock community containing 40 bacterial species. Representative sequences for each OTU were aligned using Infernal^88^ and a 16S rRNA gene phylogenetic tree was constructed using FastTree^89^. Taxonomy was assigned using BLAST^90^ and the SILVA v132 reference database^91^.

## Assessment of metabolic profiles

The capacity of the two invading species and of the resident community to utilise 31 substrates were assessed with EcoPlates (Biolog, USA). The two invading strains were first revived from glycerol stocks stored at -80 °C through 24 h of growth in 20 ml of brain heart infusion (BHI; Conda, Spain) medium at 37 °C. 20 ml of suspension was centrifuged at 5,800 rpm for 3 min, the supernatant was discarded and the cell pellet was re-suspended in 20 ml of TS buffer. The obtained suspension was diluted in TS buffer to a final OD of 0.2. This suspension was used to inoculate the EcoPlate (120 µl per well, 3 replicate wells per substrate, one plate per species). For each plate, 9 blank wells were filed with 120 µl sterile water. To assess the metabolic profile of the soil resident community, 5 g of soil were suspended in 45 ml of TS buffer (5 min of vortexing). After 10 min of decantation, the supernatant was transferred into a new tube and centrifuged 2 min at 1,000 rpm. The supernatant was used to inoculate the EcoPlate (120 µl per well). All of the plates were incubated at 25 °C without shaking and the OD was measured (590 nm; Infinite M200 PRO, Tecan, Switzerland) once a day for three days following the day of inoculation. Four replicates were performed per condition.

### Statistical analyses

All experiments were repeated four times. All statistical analyses were done in the R programming language version 4.2.1 (R Core Team 2023).

#### Invader survival

Logarithm base 10–transformation was applied to the data before analysis. Repeated measures 3-way anova was performed using the WRS2 R package^92^ to test for significant differences and interactions between disturbance treatment, invasion treatment and time. Separate models were computed for non-sterile and sterile conditions and for KP and Lm. When three-way significant interaction was recorded, robust 2-way and 1-way anova were implemented after Bonferroni correction. Cold and heat treatments were compared to the control. When significant, multiple pairwise comparisons were performed after Bonferroni adjustments to determine which treatment means were different.

#### Metabolic profiles

To analyse the data obtained from the Ecoplates, OD values of the three replicates were averaged at the three time points for each substrate and blanks. Average OD values of substrates were compared to the average ODs recorded on blanks. A substrate was considered metabolised when its average OD exceeded the blank average OD by at least 0.1 OD unit at day 2 and day 3. In this case, the maximum OD was kept for quantitative analysis since it represents the maximum amount of substrate consumed. For non-metabolized substrates, the value was set to zero. In order to compare substrates’ utilisation between Kp, Lm and the native soil community, differences between maximum ODs were analysed by one-way anova for each substrate. When differences were significant, pairwise comparisons between Kp, Lm and the native soil community metabolic capacities were assessed via Tukey HSD tests (*p* < 0.05).

#### Bacterial diversity

We used three diversity metrics to describe the structure of microbial communities: Faith’s Phylogenetic Diversity^93^, species richness (observed species) and evenness (Simpson’s reciprocal index, equitability) were calculated based on rarefied OTU tables (20,000 sequences per sample). UniFrac distance matrices^94,95^ were also computed to detect global variations in the composition of microbial communities. DNA sequences from the two invading bacteria were excluded from the dataset before analyses to prevent any distortion due to their high number of sequences at T0.

All diversity data is available in the NCBI Sequence Read Archive under the accession number PRJNA884273.

At each timepoint (T0, T20 and T40), 2-ways anovas were performed to evaluate the effects of the disturbance regimen (control, cold and heat), the invasion treatment (No invasion, Invasion with either *L. monocytogenes* or *K. pneumoniae*, and Co-invasion) and their interaction on bacterial diversity indices (Observed Species, Simpson’s reciprocal index and Faith’s Phylogenetic Diversity). Pairwise comparisons were assessed via Tukey HSD tests (*p* < 0.05).

We also assessed the effects of the disturbance regimen, the invasion treatment, the time of sampling, as well as interactions between these factors, on bacterial community structure using the weighted UniFrac distance matrix with permanovas (*adonis2* function, vegan R package). Pairwise comparisons between levels of a given factor were performed using the *pairwise.adonis()* function with the BH adjustment method for multiple comparisons, within the pairwiseAdonis R package^96^. Note that sequences specifically assigned either to listeriaceae and klebsiellaceae families were removed prior to analyses to avoid distorting the results.

#### Detection of potentially permissive/competitive *OTUs*

An indicator species analysis^97^ was conducted to detect OTUs whose abundance was significantly increased/decreased in experimental conditions where *L. monocytogenes* or *K. pneumoniae* were more pervasive. For Lm, cold-treated and control microcosms were considered as permissive while the permissive microcosms were the heat-treated and control ones for Kp. The *multipatt()* function was used to detect OTUs displaying significant increased or decreased abundances in permissive conditions based on the calculation of an extension of the original Indicator Value method^98^. Significance of the associations is then tested using a permutation test (*p* < 0.05). OTUs for which significant associations were detected were then plotted through heatmaps.

### Supplementary Information

Supplementary Information.

Supplementary Information.

## Data Availability

All diversity data is available in the NCBI Sequence Read Archive under the accession number PRJNA884273 (https://www.ncbi.nlm.nih.gov/search/all/?term=PRJNA884273).
