# Supplementary material for: Idiosyncratic invasion trajectories of human bacterial pathogens facing temperature disturbances in soil microbial communities
Source: Sci Rep. 2024 May 29;14:12375. doi: 10.1038/s41598-024-63284-5 (PMC11137084; doi:10.1038/s41598-024-63284-5)
Supplement: Supplementary file 1 — Supplementary Information. [file 41598_2024_63284_MOESM1_ESM.pdf]

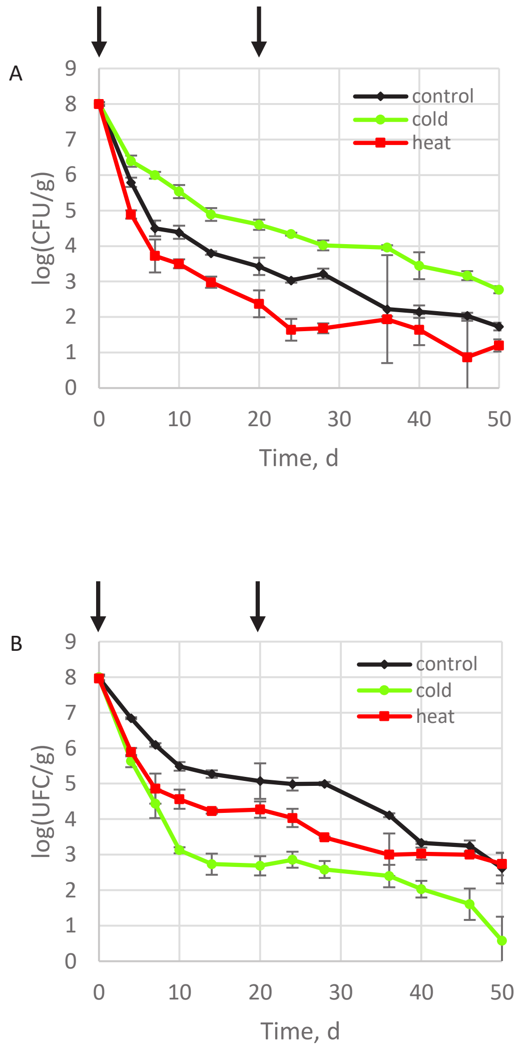

Figure S1. Fate of the population of *L. monocytogenes* L9 (A) and *K. pneumoniae* MGH 78578 (B) during co-invasion of soil microcosms. Arrows indicate the time when disturbance was applied.

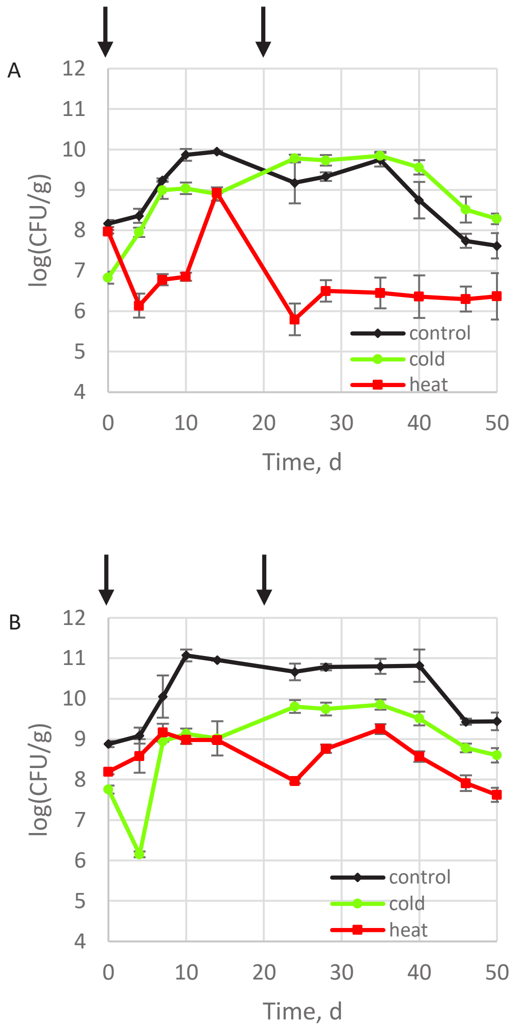

Figure S2. Fate of the population of *L. monocytogenes* L9 (A) and *K. pneumoniae* MGH 78578 (B) during co- invasion of sterilised soil microcosms. Arrows indicate the time when disturbance was applied.

A

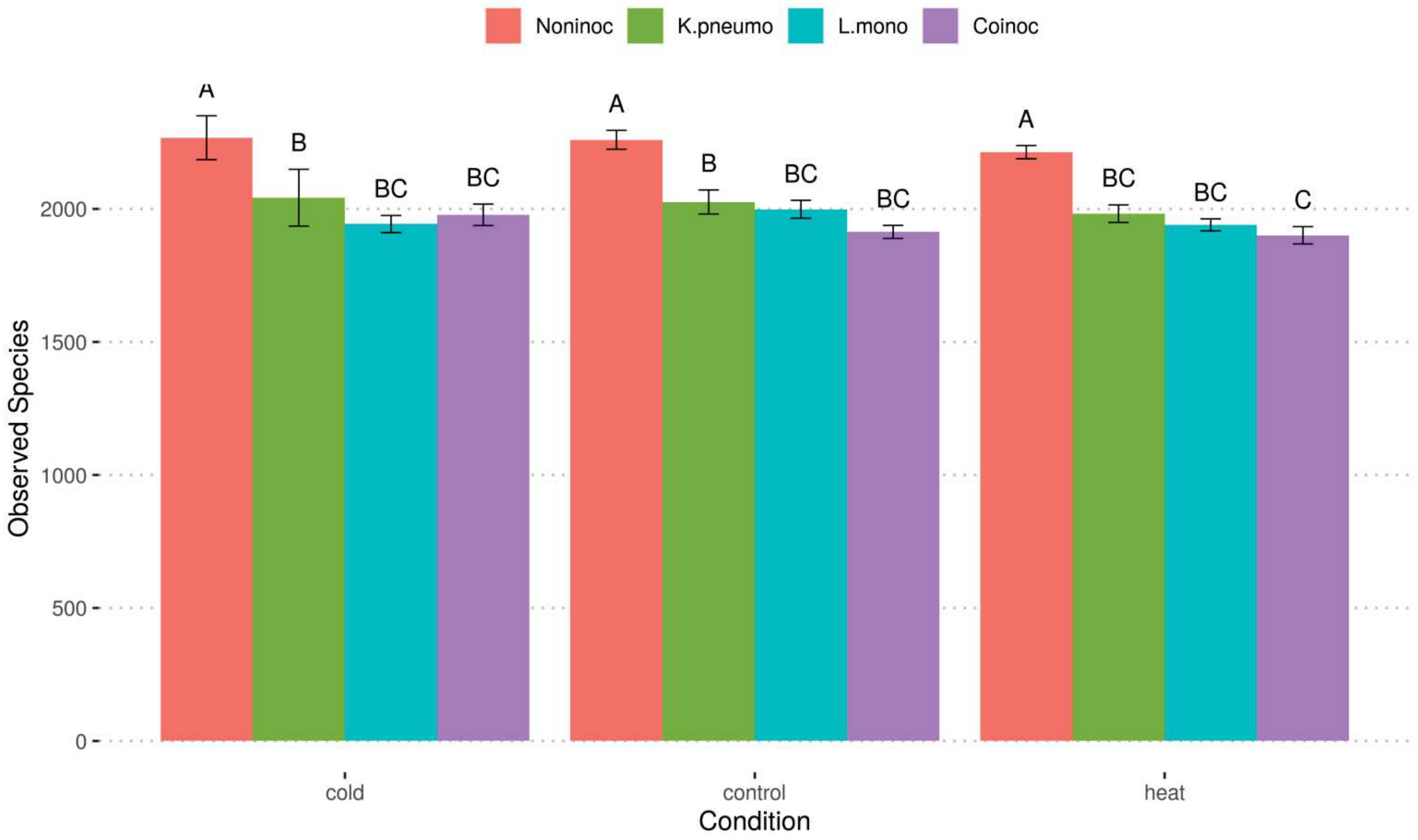

Figure S3. Observed species metrics according to treatments and time of incubation. (A) time 0; (B) time 20; (C) time 40.

B

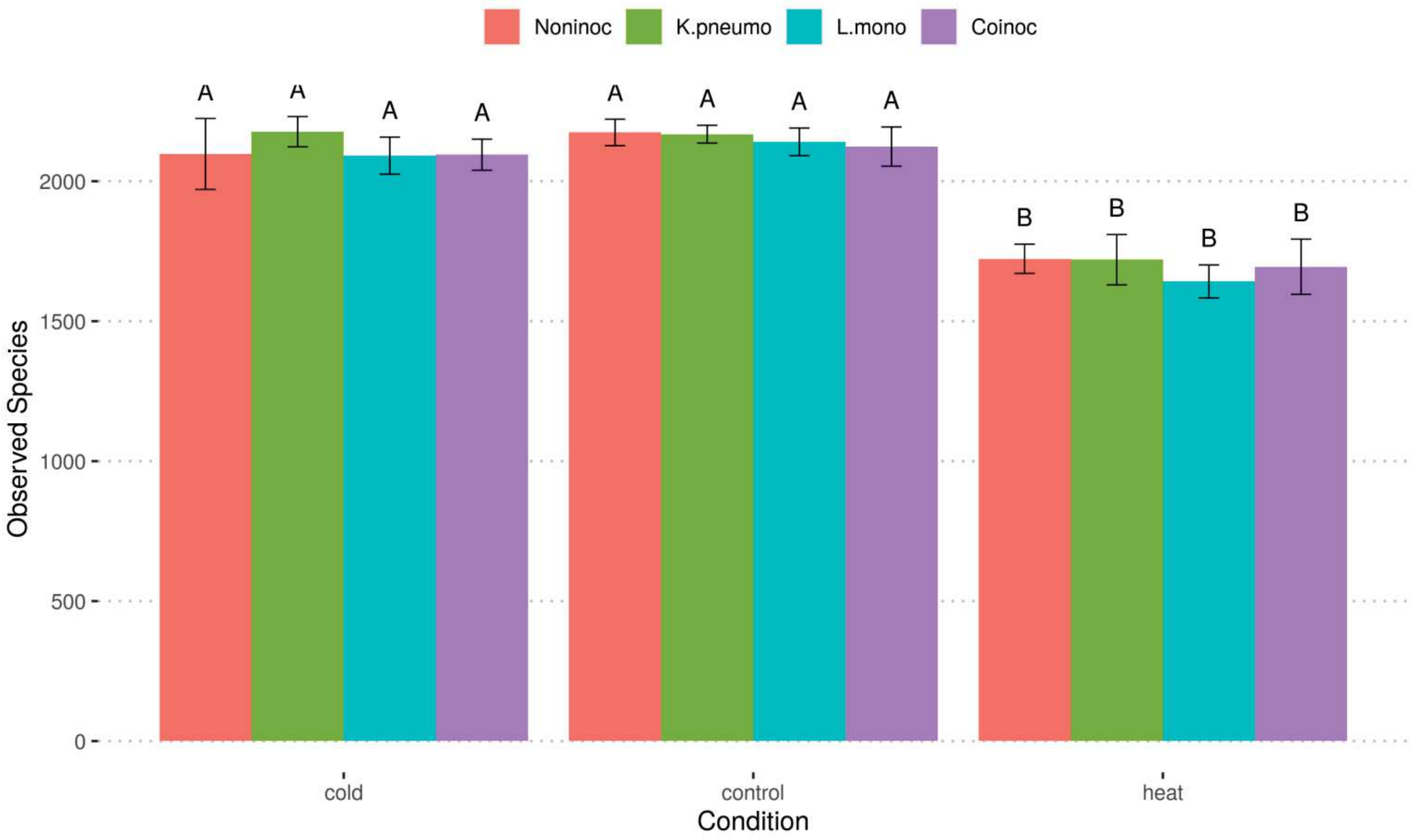

Figure S3. Observed species metrics according to treatments and time of incubation. (A) time 0; (B) time 20; (C) time 40.

C

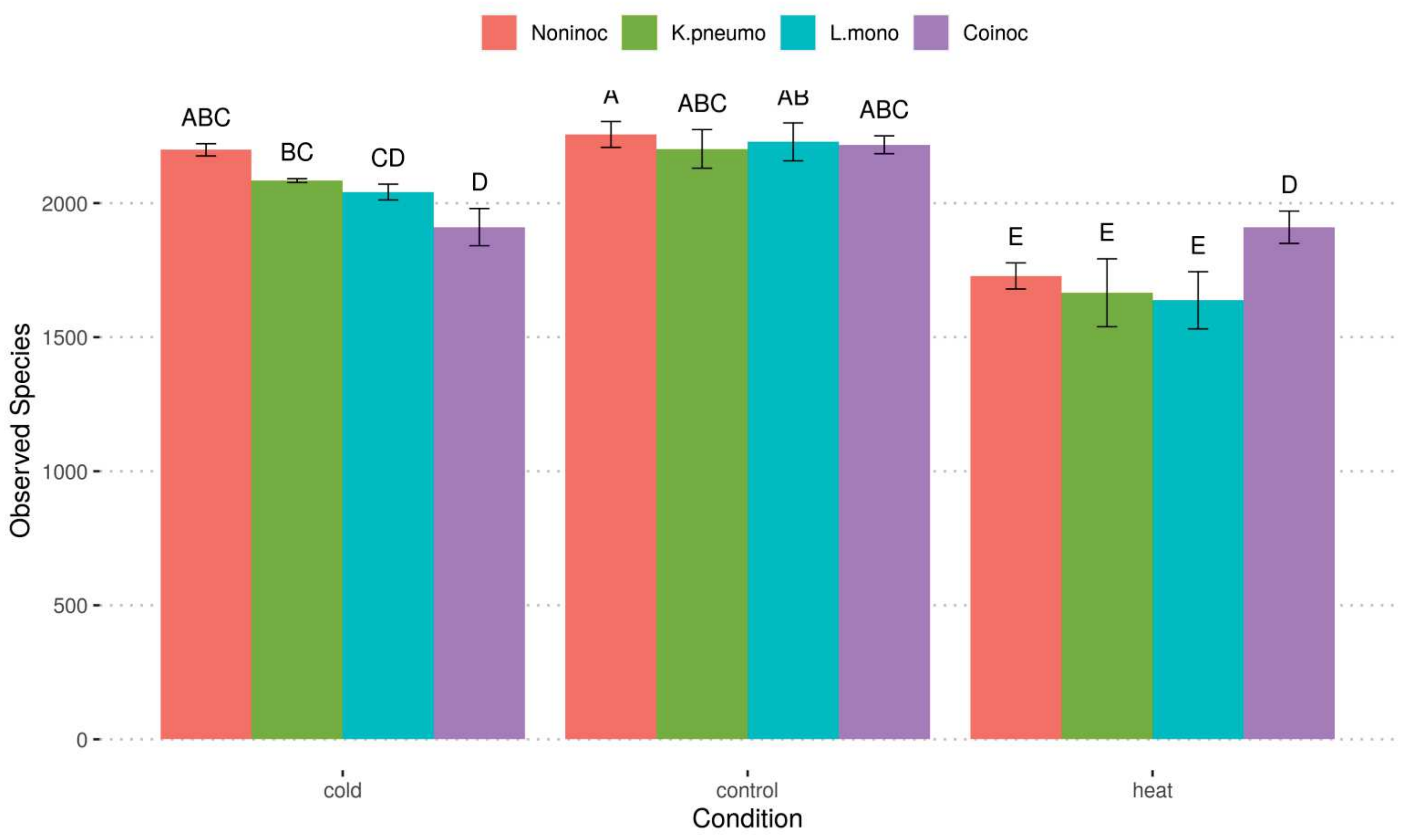

Figure S3. Observed species metrics according to treatments and time of incubation. (A) time 0; (B) time 20; (C) time 40.

A

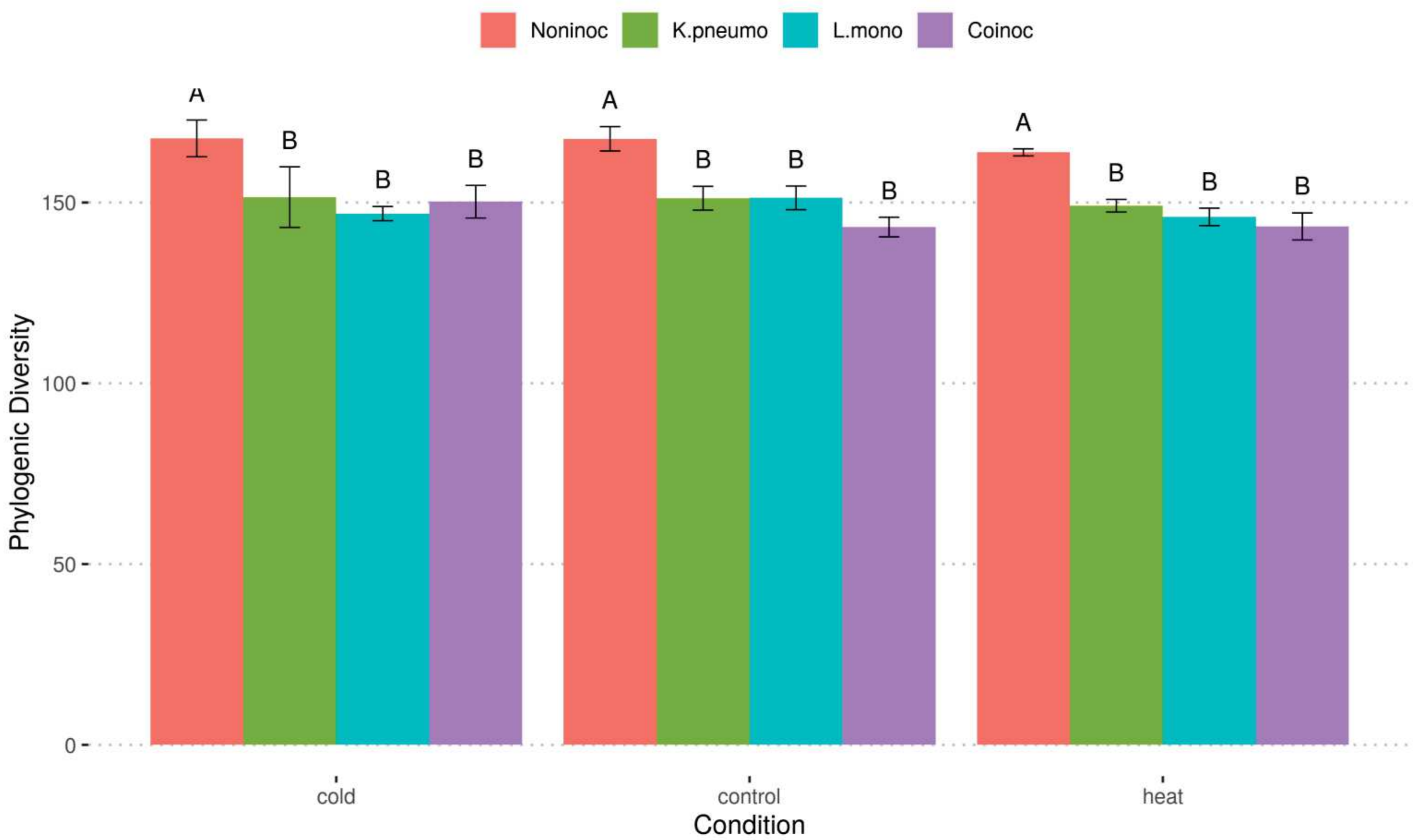

Figure S4. Phylogenetic metrics according to treatments and time of incubation. (A) time 0; (B) time 20; (C) time 40.

B

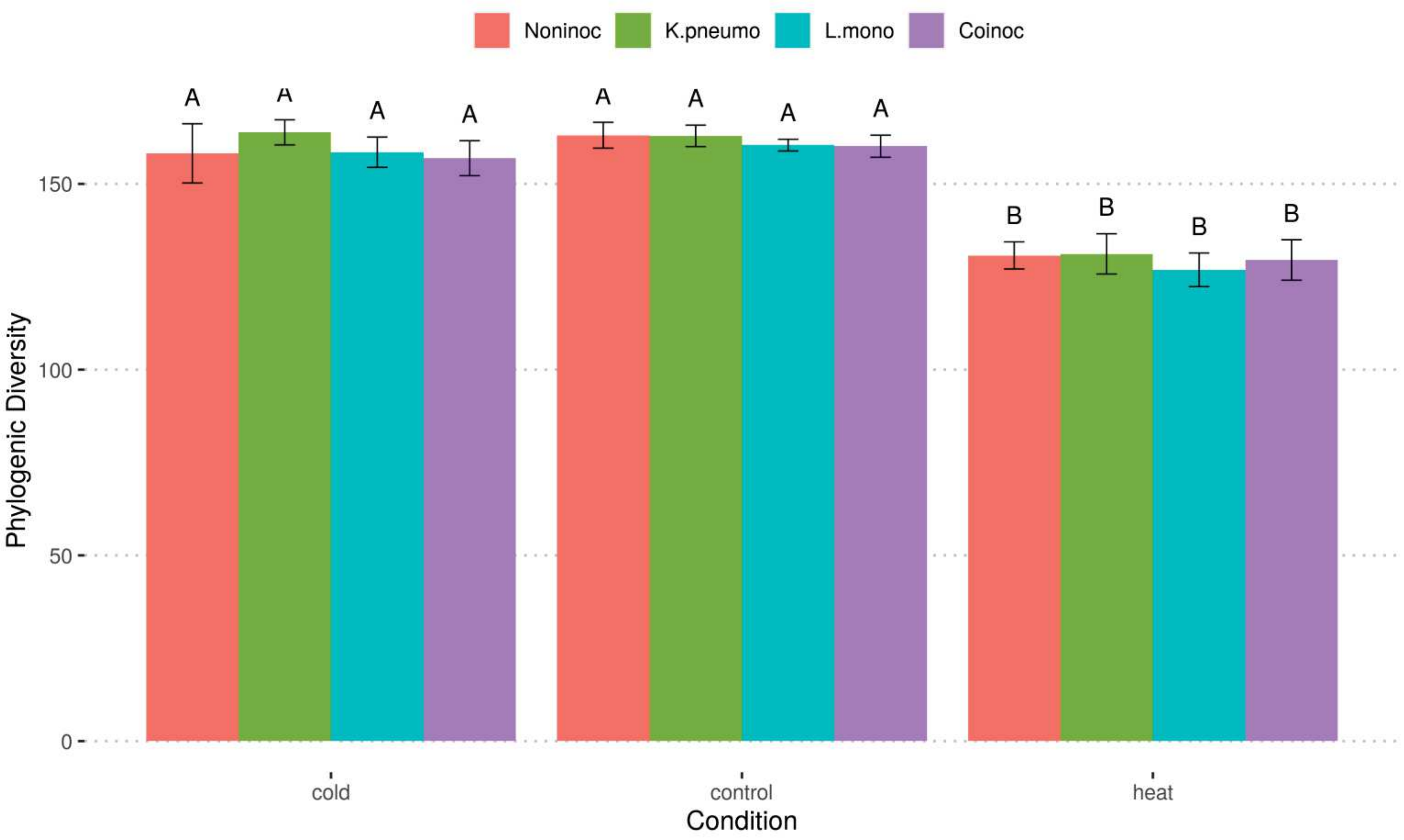

Figure S4. Phylogenetic metrics according to treatments and time of incubation. (A) time 0; (B) time 20; (C) time 40.

C

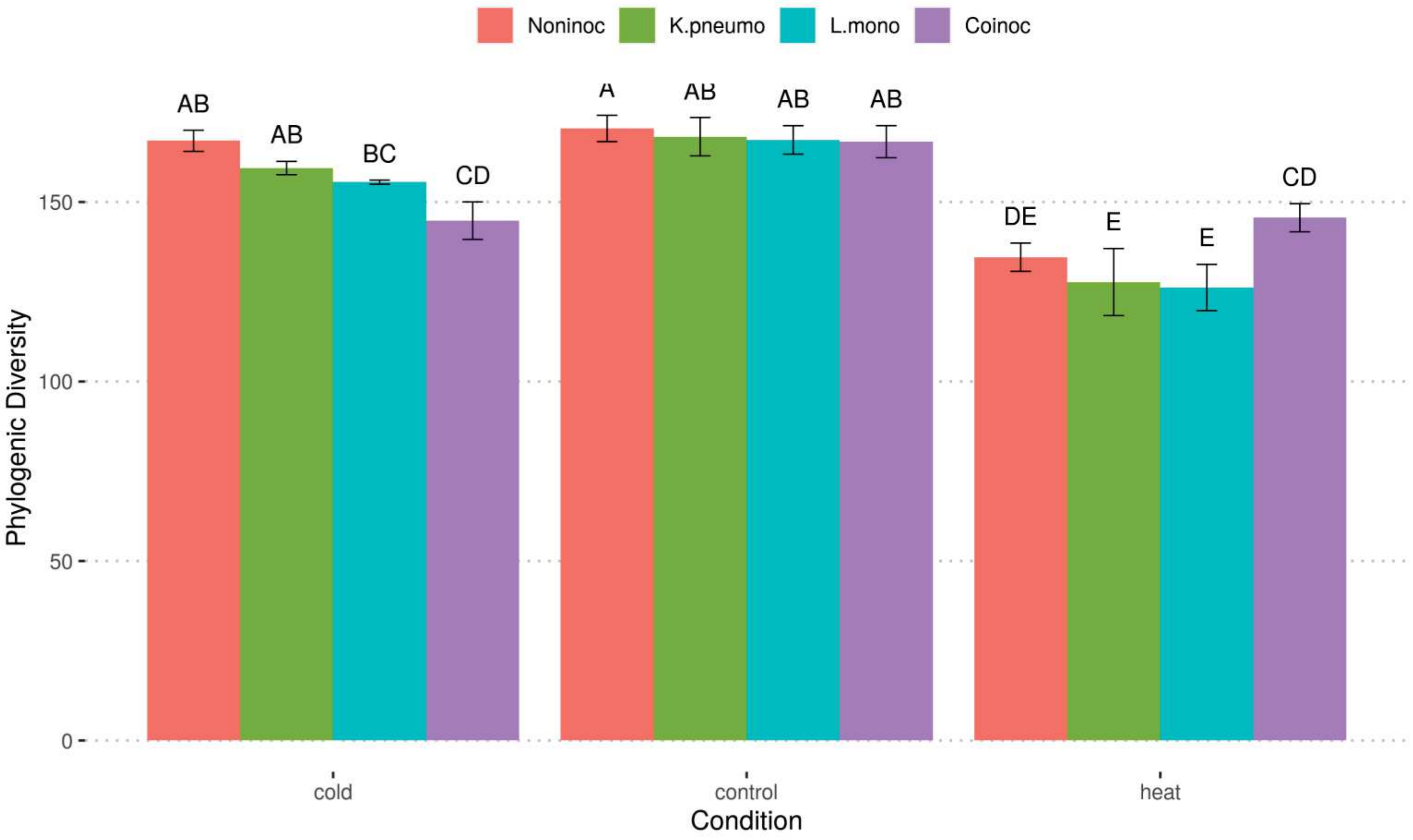

Figure S4. Phylogenetic metrics according to treatments and time of incubation. (A) time 0; (B) time 20; (C) time 40.

A

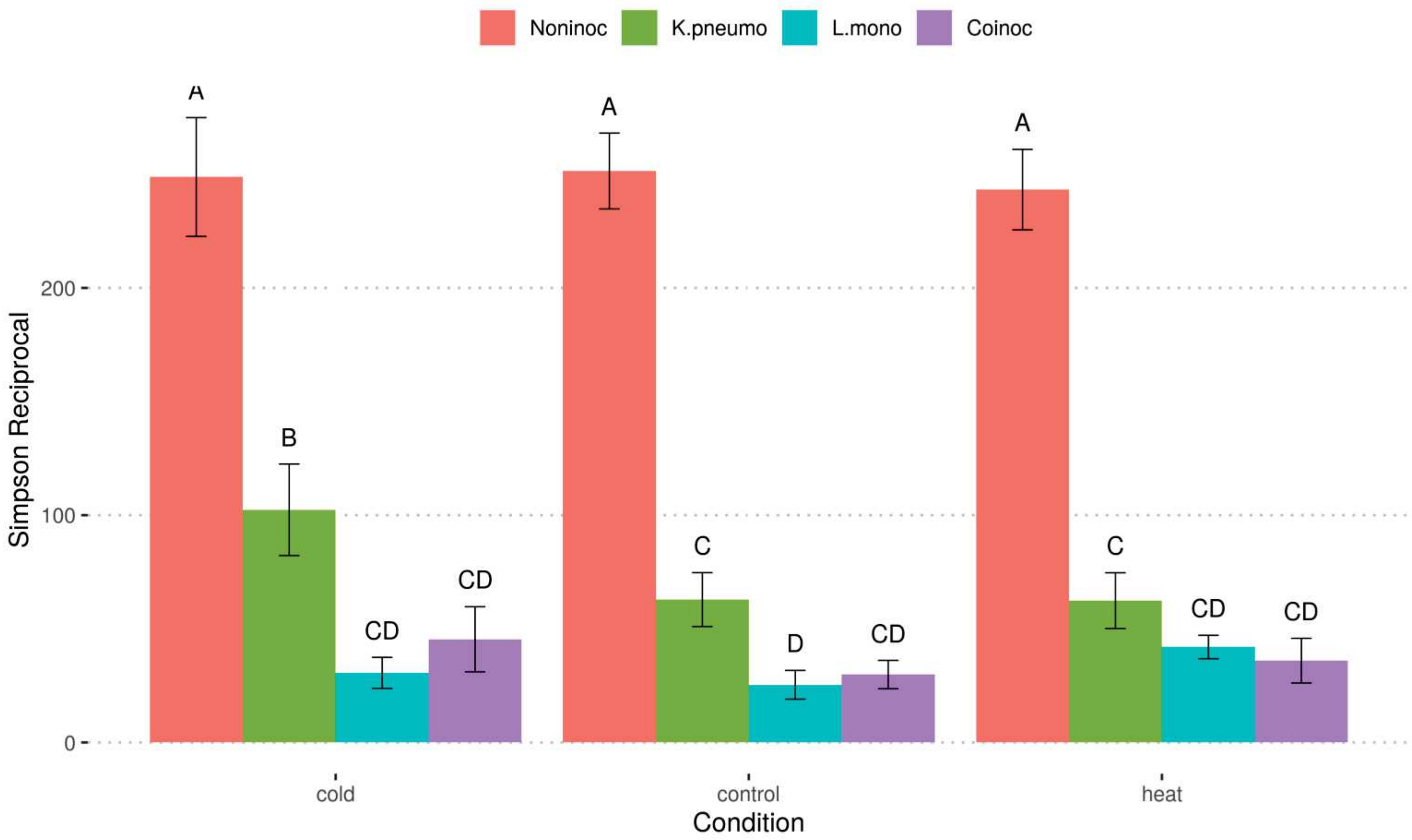

Figure S5. Simpson reciprocal metrics according to treatments and time of incubation. (A) time 0; (B) time 20; (C) time 40.

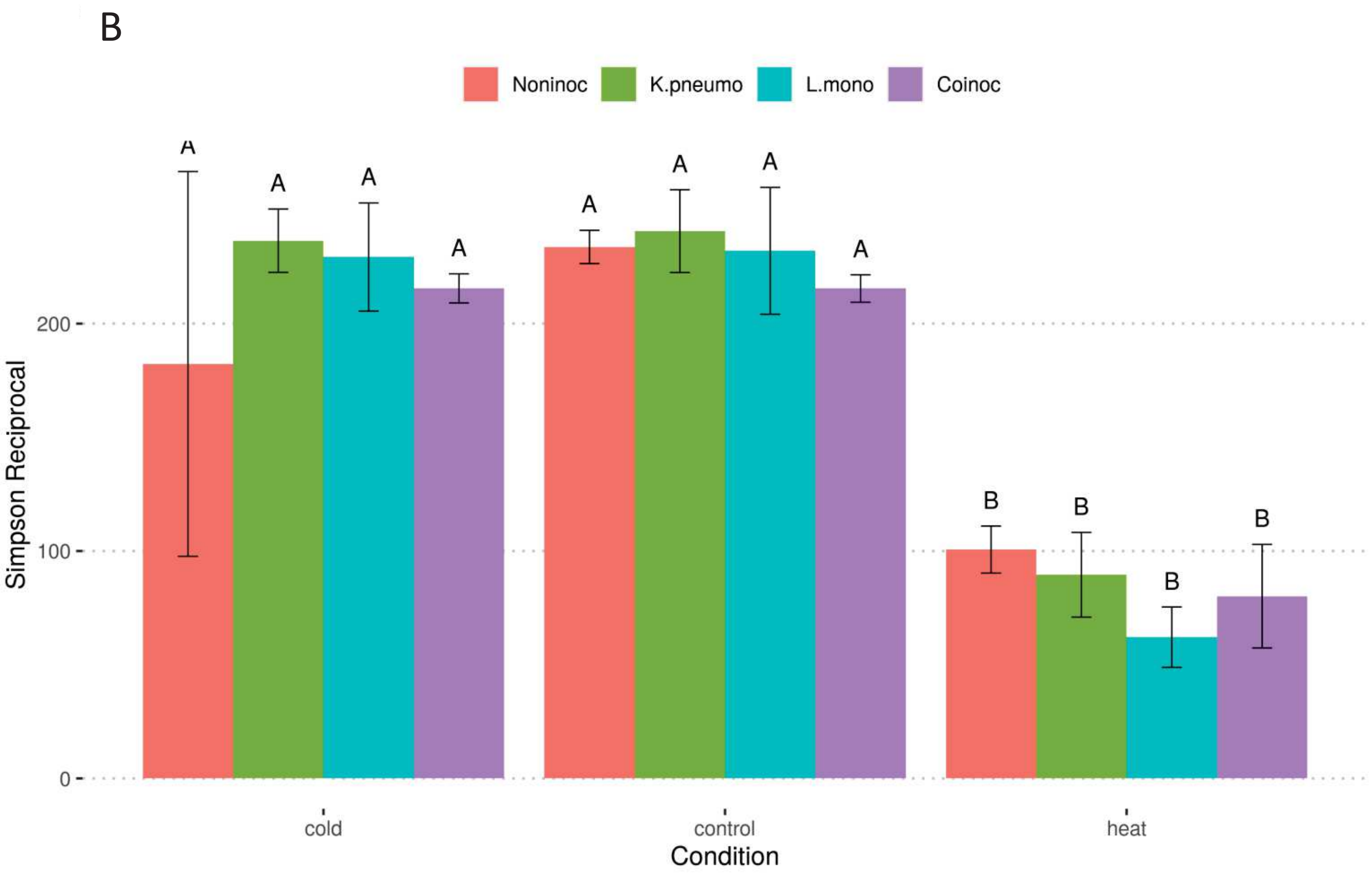

Figure S5. Simpson reciprocal metrics according to treatments and time of incubation. (A) time 0; (B) time 20; (C) time 40.

C

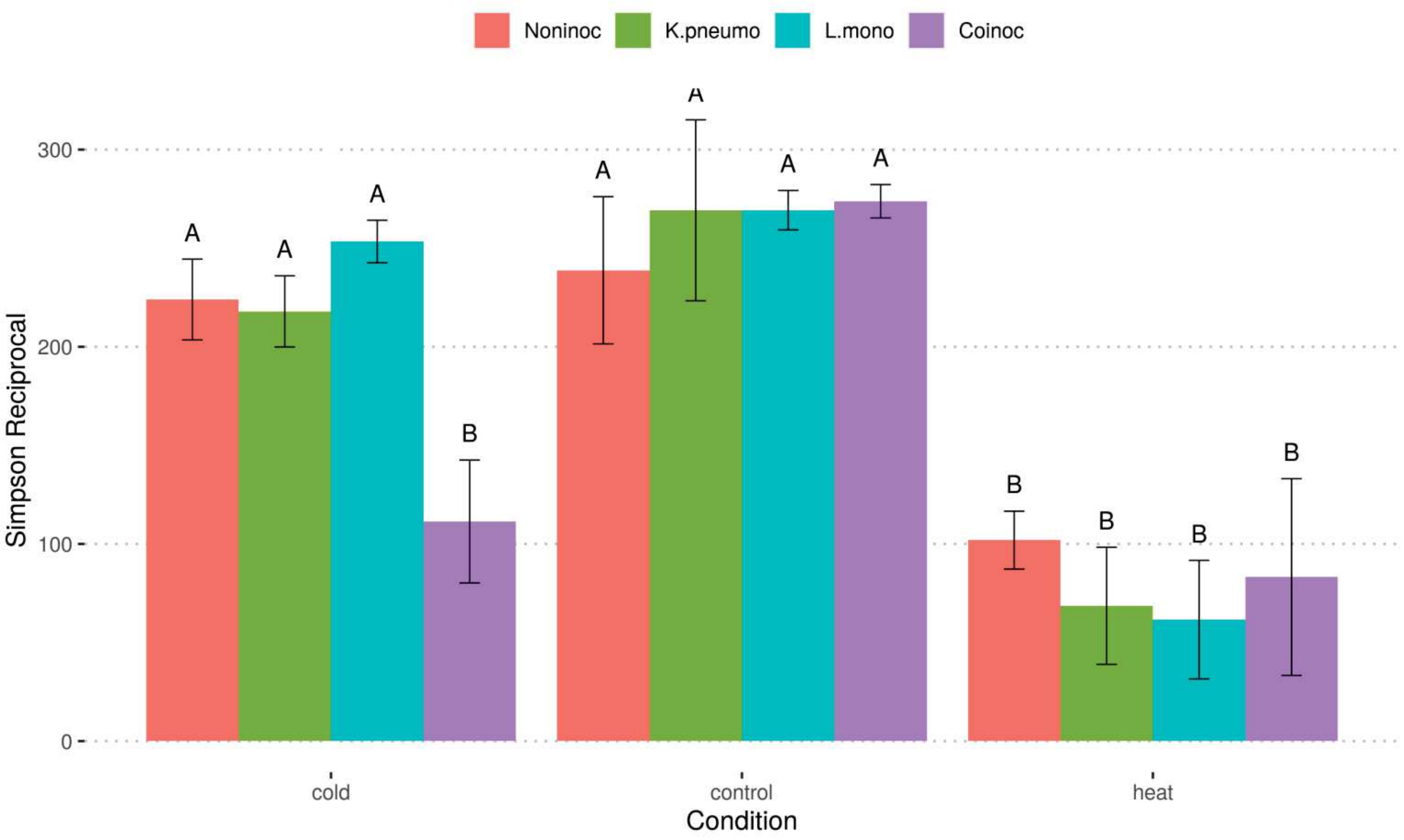

Figure S5. Simpson reciprocal metrics according to treatments and time of incubation. (A) time 0; (B) time 20; (C) time 40.

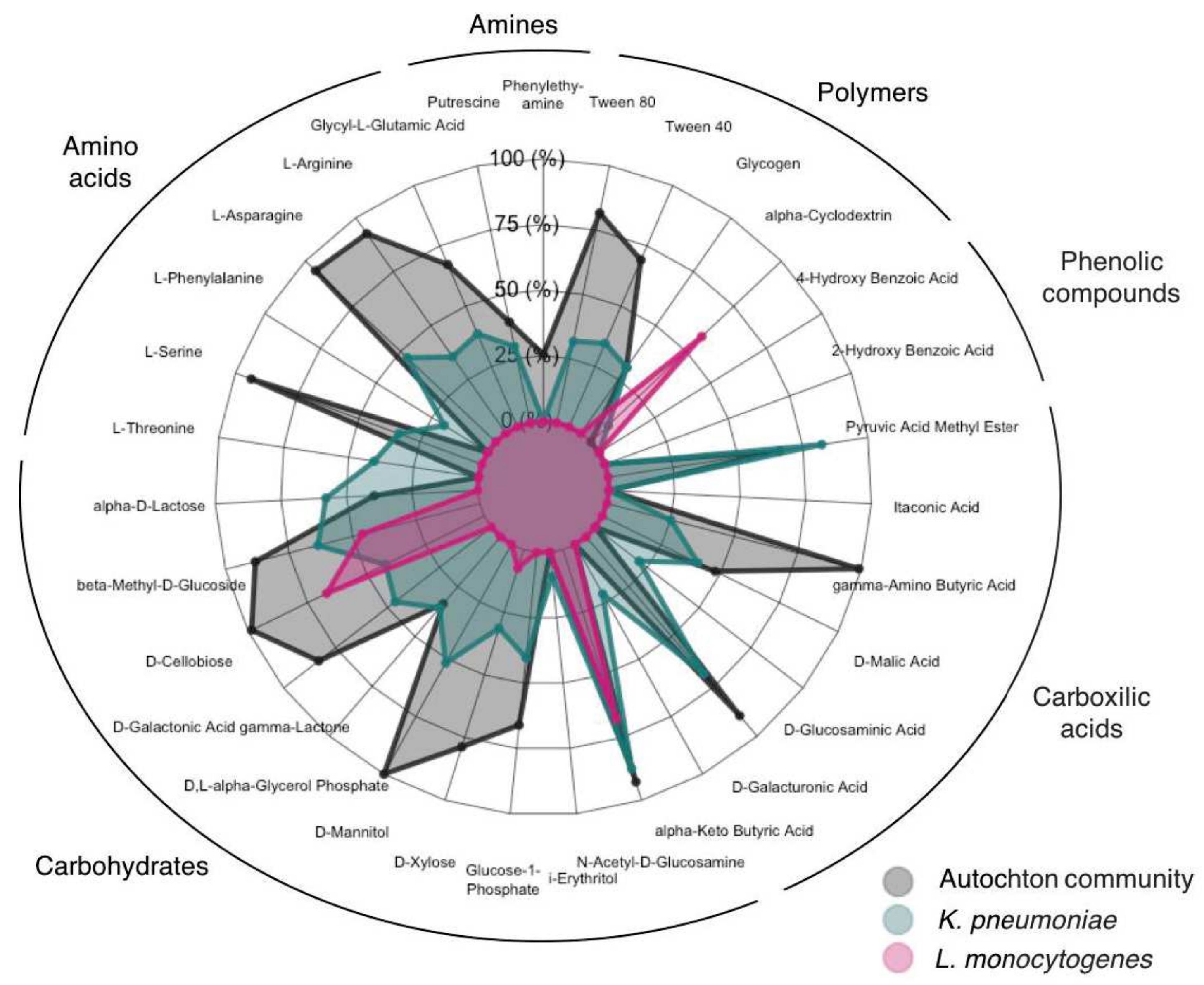

Figure S6. Estimation of niche breadth of the autochthonous soil communities (grey) and allochthon strains *Klebsiella pneumoniae* MGH 78578 (green) and *Listeria monocytogenes* L9 (pink). The ability of the autochthon community and of the allochthon strains to metabolize 31 substrates was assessed by measuring the optical density produced by the reduction of a tetrazolium dye during the metabolization (EcoPlates, Biolog). The results are shown in percentage with 100% corresponding to the highest optical density recorded in the experiment.

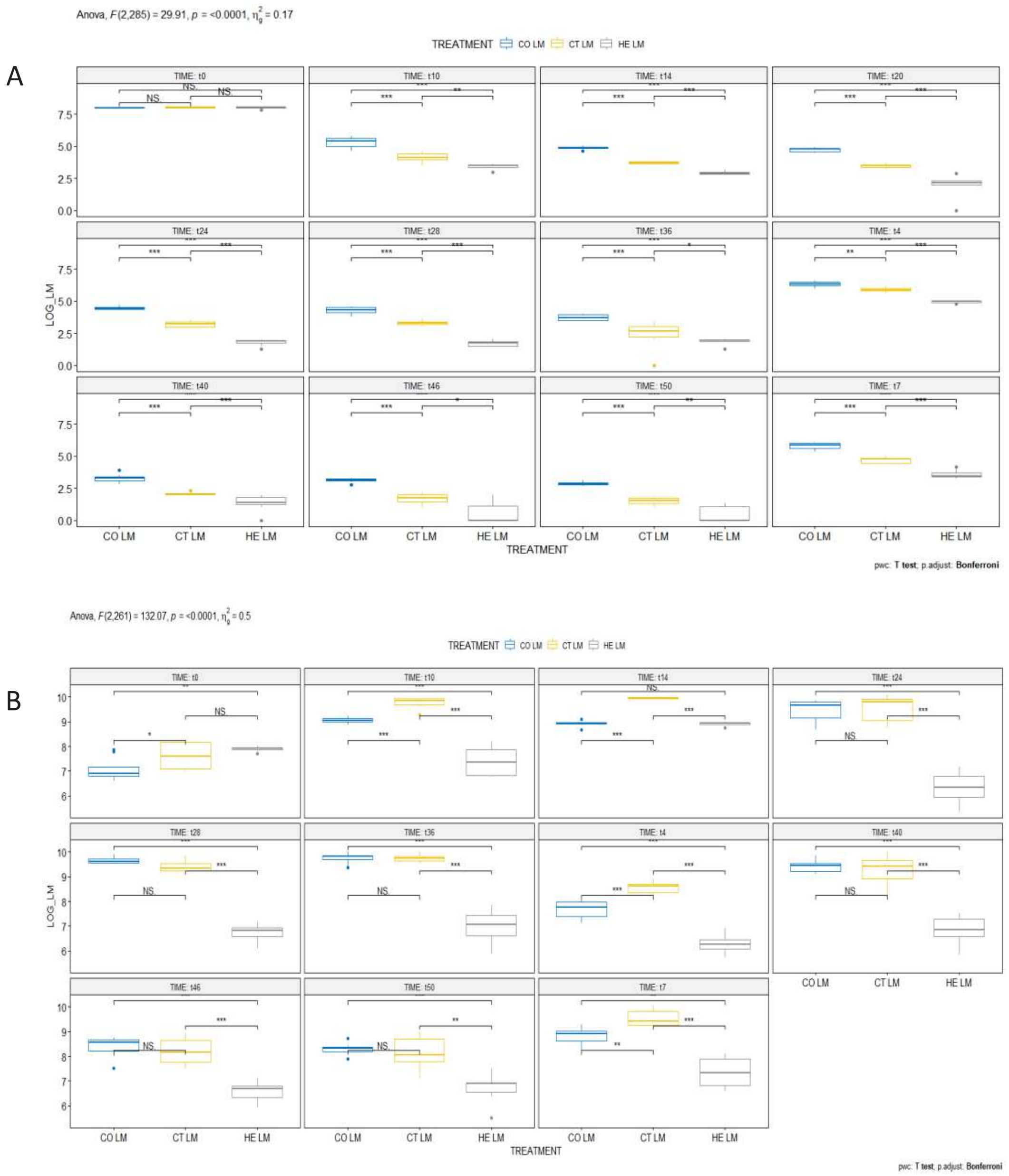

Figure S7. Boxplot of the log-transformed population of *L. monocytogenes* L9 in non sterile (A) and sterilised (B) soil microcosms. NS: non significant. The level of significance of the differences is recorded with stars.

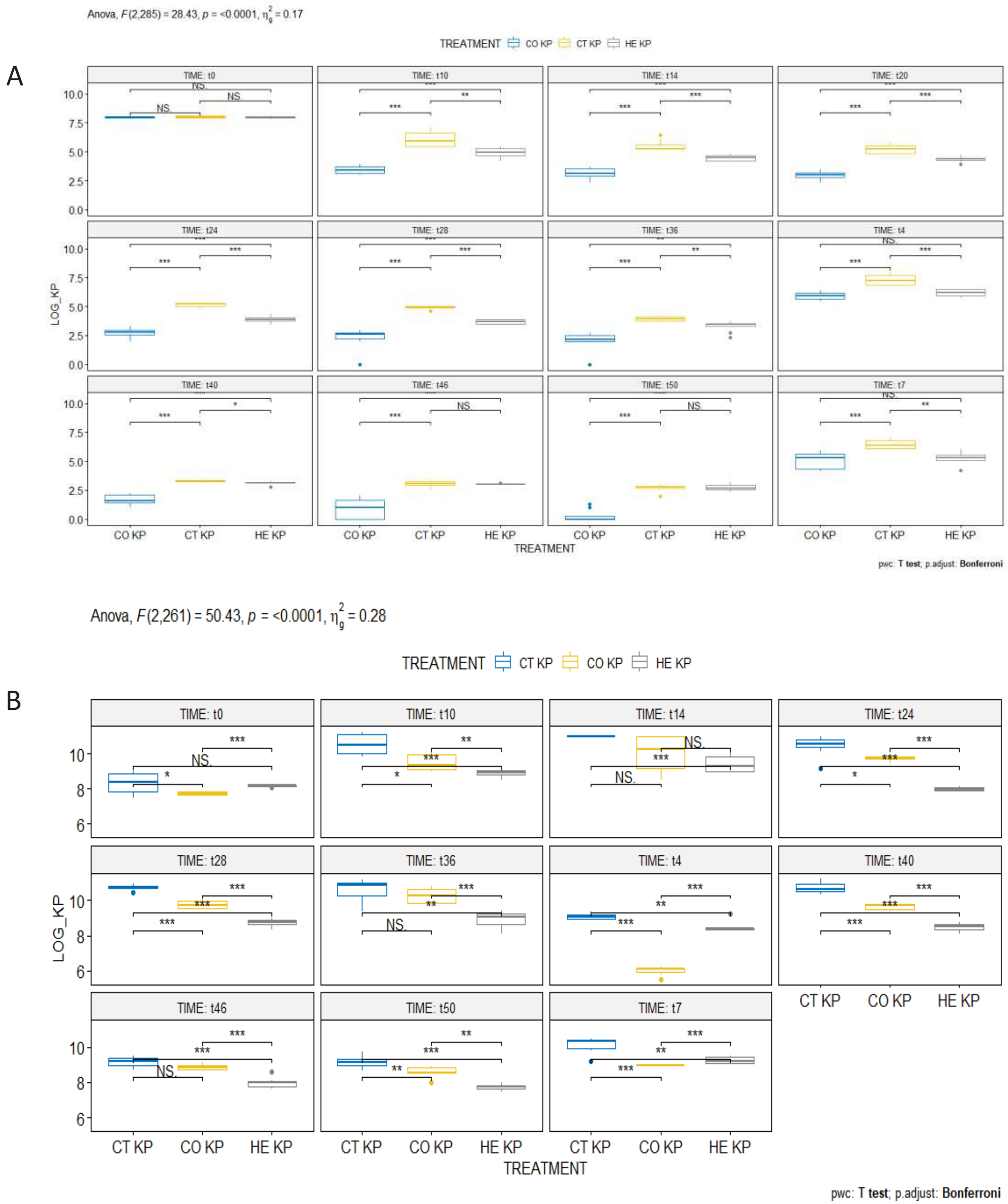

Figure S8. Boxplot of the log-transformed population of *K. pneumoniae* MGH 78578 in non sterile (A) and sterilised (B) soil microcosms. NS: non significant. The level of significance of the differences is recorded with stars.

TABLE S1

OTU.ID	Phylum	Genus	CumAb	Permissive
OTU-45381	Acidobacteria	Blastocatella	388	-
OTU-1	Acidobacteria	Bryobacter	4528	-
OTU-120	Acidobacteria	Bryobacter	1393	-
OTU-20	Acidobacteria	Bryobacter	986	-
OTU-4758	Acidobacteria	Unassigned	636	-
OTU-444	Actinobacteri	Gaiella	1094	-
OTU-16938	Actinobacteri	Luedemannell	195	-
OTU-1131	Actinobacteri	uncultured ba	1771	-
OTU-229	Actinobacteri	uncultured ba	538	-
OTU-54806	Actinobacteri	uncultured ba	250	-
OTU-274	Actinobacteri	uncultured Ru	1556	-
OTU-41	Actinobacteri	uncultured soi	2199	-
OTU-76	Bacteroidetes	Flavisolibacter	5533	-
OTU-2776	Bacteroidetes	Flavisolibacter	4579	-
OTU-90430	Bacteroidetes	Flavisolibacter	641	-
OTU-1757	Bacteroidetes	Flavisolibacter	463	-
OTU-466	Bacteroidetes	Flavisolibacter	397	-
OTU-82	Bacteroidetes	Segetibacter	774	-
OTU-23439	Bacteroidetes	Unassigned	574	-
OTU-83	Bacteroidetes	uncultured ba	280	-
OTU-509	Chloroflexi	uncultured	616	-
OTU-89900	Firmicutes	Bacillus	760	-
OTU-2476	Firmicutes	Bacillus	524	-
OTU-22178	Firmicutes	Clostridium se	332	-
OTU-23750	Firmicutes	Tumebacillus	129	-
OTU-25718	Firmicutes	uncultured	415	-
OTU-476	Firmicutes	uncultured ba	481	-
OTU-191	Gemmatimon	Gemmatimon	477	-
OTU-15	Proteobacteri	Lysobacter	6674	-
OTU-74	Proteobacteri	Lysobacter	457	-
OTU-608	Proteobacteri	Lysobacter	352	-
OTU-25887	Proteobacteri	Massilia	2580	-
OTU-30556	Proteobacteri	Massilia	561	-
OTU-46573	Proteobacteri	Massilia	499	-
OTU-56	Proteobacteri	Methylocystis	1477	-
OTU-132	Proteobacteri	Microvirga	1231	-
OTU-14419	Proteobacteri	Microvirga	612	-
OTU-127	Proteobacteri	Nitrosospira	914	-
OTU-3	Proteobacteri	Noviherbaspir	14867	-
OTU-62	Proteobacteri	Skermanella	3251	-
OTU-14760	Proteobacteri	Sphingomona	1113	-
OTU-25582	Proteobacteri	Sphingomona	1025	-
OTU-257882	Proteobacteri	Sphingomona	365	-
OTU-106319	Proteobacteri	Sphingomona	310	-
OTU-51	Proteobacteri	Unassigned	4771	-
OTU-92	Proteobacteri	Unassigned	4230	-
OTU-13146	Proteobacteri	Unassigned	496	-
OTU-21	Proteobacteri	Unassigned	228	-

OTU-4291	Proteobacteri	uncultured	311	-
OTU-1138	Proteobacteri	uncultured Ar	597	-
OTU-13940	Acidobacteria	Blastocatella	161	+
OTU-212	Acidobacteria	Candidatus Ko	705	+
OTU-89	Acidobacteria	Candidatus So	2941	+
OTU-256471	Acidobacteria	Candidatus So	1246	+
OTU-124	Acidobacteria	Candidatus So	748	+
OTU-380	Acidobacteria	Candidatus So	511	+
OTU-82772	Acidobacteria	Candidatus So	293	+
OTU-1117	Acidobacteria	Candidatus So	292	+
OTU-152453	Acidobacteria	metagenome	256	+
OTU-376	Acidobacteria	Occallatibacte	619	+
OTU-288	Acidobacteria	Paludibaculum	257	+
OTU-33	Acidobacteria	RB41	5105	+
OTU-24	Acidobacteria	RB41	2547	+
OTU-283	Acidobacteria	RB41	1831	+
OTU-7771	Acidobacteria	RB41	362	+
OTU-1036	Acidobacteria	Subgroup 10	411	+
OTU-45	Acidobacteria	Unassigned	2368	+
OTU-196	Acidobacteria	Unassigned	1318	+
OTU-49	Acidobacteria	Unassigned	857	+
OTU-684	Acidobacteria	Unassigned	834	+
OTU-30	Acidobacteria	Unassigned	758	+
OTU-463	Acidobacteria	Unassigned	744	+
OTU-55	Acidobacteria	Unassigned	669	+
OTU-555	Acidobacteria	Unassigned	484	+
OTU-20663	Acidobacteria	Unassigned	433	+
OTU-87	Acidobacteria	Unassigned	390	+
OTU-4322	Acidobacteria	Unassigned	387	+
OTU-154895	Acidobacteria	Unassigned	369	+
OTU-233	Acidobacteria	Unassigned	367	+
OTU-460	Acidobacteria	Unassigned	319	+
OTU-113001	Acidobacteria	Unassigned	301	+
OTU-190893	Acidobacteria	Unassigned	291	+
OTU-334	Acidobacteria	Unassigned	290	+
OTU-3623	Acidobacteria	Unassigned	276	+
OTU-297	Acidobacteria	Unassigned	270	+
OTU-735	Acidobacteria	Unassigned	247	+
OTU-814	Acidobacteria	Unassigned	242	+
OTU-47	Acidobacteria	uncultured	2866	+
OTU-571	Acidobacteria	uncultured Ac	1406	+
OTU-336	Acidobacteria	uncultured Ac	1065	+
OTU-1820	Acidobacteria	uncultured Ac	643	+
OTU-1429	Acidobacteria	uncultured Ac	545	+
OTU-128145	Acidobacteria	uncultured Ac	445	+
OTU-123	Acidobacteria	uncultured ba	1964	+
OTU-245	Acidobacteria	uncultured ba	1593	+
OTU-54	Acidobacteria	uncultured ba	1417	+
OTU-6813	Acidobacteria	uncultured ba	1120	+
OTU-462	Acidobacteria	uncultured ba	592	+

OTU-561	Acidobacteria	uncultured ba	435	+
OTU-397	Acidobacteria	uncultured ba	429	+
OTU-16486	Acidobacteria	uncultured ba	380	+
OTU-3354	Acidobacteria	uncultured ba	365	+
OTU-188	Acidobacteria	uncultured ba	324	+
OTU-2066	Acidobacteria	uncultured ba	308	+
OTU-3191	Actinobacteria	Actinoplanes	196	+
OTU-29	Actinobacteria	Arthrobacter	3356	+
OTU-4166	Actinobacteria	Ilumatobacter	205	+
OTU-84	Actinobacteria	Kribbella	810	+
OTU-119	Actinobacteria	Leifsonia	1231	+
OTU-1697	Actinobacteria	Microlunatus	219	+
OTU-31	Actinobacteria	Nakamurella	300	+
OTU-652	Actinobacteria	Nocardioides	641	+
OTU-264	Actinobacteria	Nocardioides	327	+
OTU-441	Actinobacteria	Nocardioides	308	+
OTU-1573	Actinobacteria	Nocardioides	205	+
OTU-13099	Actinobacteria	Nocardioides	192	+
OTU-4520	Actinobacteria	Oerskovia	244	+
OTU-32998	Actinobacteria	Pseudonocard	261	+
OTU-208	Actinobacteria	Solirubrobacte	435	+
OTU-121	Actinobacteria	Unassigned	541	+
OTU-308	Actinobacteria	uncultured	489	+
OTU-388	Armatimonad	Chthonomona	303	+
OTU-159143	Bacteroidetes	Adhaeribacter	482	+
OTU-286	Bacteroidetes	Adhaeribacter	273	+
OTU-145563	Bacteroidetes	Adhaeribacter	132	+
OTU-324	Bacteroidetes	Chryseolinea	631	+
OTU-187	Bacteroidetes	Ferruginibacte	657	+
OTU-8461	Bacteroidetes	Ferruginibacte	498	+
OTU-12821	Bacteroidetes	Ferruginibacte	472	+
OTU-1569	Bacteroidetes	Ferruginibacte	337	+
OTU-71666	Bacteroidetes	Ferruginibacte	219	+
OTU-194	Bacteroidetes	Ferruginibacte	168	+
OTU-991	Bacteroidetes	Flavitalea	370	+
OTU-3215	Bacteroidetes	Flavobacteriu	447	+
OTU-556	Bacteroidetes	Flavobacteriu	437	+
OTU-186114	Bacteroidetes	Flavobacteriu	340	+
OTU-80054	Bacteroidetes	Flavobacteriu	77	+
OTU-275	Bacteroidetes	Mucilaginibac	110	+
OTU-5787	Bacteroidetes	Niastella	214	+
OTU-60	Bacteroidetes	Parafilimonas	617	+
OTU-890	Bacteroidetes	Unassigned	309	+
OTU-116646	Bacteroidetes	uncultured	1994	+
OTU-105	Bacteroidetes	uncultured	1555	+
OTU-346	Bacteroidetes	uncultured	1121	+
OTU-3288	Bacteroidetes	uncultured	425	+
OTU-2167	Bacteroidetes	uncultured	422	+
OTU-11796	Bacteroidetes	uncultured	272	+
OTU-908	Bacteroidetes	uncultured	255	+

OTU-1936	Bacteroidetes	uncultured	235	+
OTU-291	Bacteroidetes	uncultured	175	+
OTU-36	Bacteroidetes	uncultured	151	+
OTU-2774	Bacteroidetes	uncultured ba	433	+
OTU-502	Bacteroidetes	uncultured Ba	384	+
OTU-255	Chloroflexi	uncultured	516	+
OTU-680	Chloroflexi	uncultured ba	186	+
OTU-1067	Fibrobacteres	possible genus	268	+
OTU-235	Gemmatimon	Gemmatimona	951	+
OTU-1116	Gemmatimon	Gemmatimona	371	+
OTU-90	Gemmatimon	uncultured	4634	+
OTU-3090	Gemmatimon	uncultured	1280	+
OTU-1760	Gemmatimon	uncultured	1103	+
OTU-361	Gemmatimon	uncultured	756	+
OTU-277	Gemmatimon	uncultured	241	+
OTU-7	Nitrospirae	Nitrospira	1631	+
OTU-253	Nitrospirae	Nitrospira	1009	+
OTU-171	Proteobacteria	Acidibacter	1344	+
OTU-197	Proteobacteria	Altererythroba	476	+
OTU-100	Proteobacteria	Arenimonas	1798	+
OTU-89519	Proteobacteria	Arenimonas	188	+
OTU-11	Proteobacteria	Bradyrhizobiu	7452	+
OTU-1641	Proteobacteria	Devosia	149	+
OTU-70	Proteobacteria	Dokdonella	246	+
OTU-575	Proteobacteria	Dongia	162	+
OTU-23	Proteobacteria	Lysobacter	5106	+
OTU-381	Proteobacteria	Nordella	260	+
OTU-1778	Proteobacteria	Pelomonas	421	+
OTU-161	Proteobacteria	Pseudomonas	1728	+
OTU-1817	Proteobacteria	Reyranella	686	+
OTU-11627	Proteobacteria	Reyranella	264	+
OTU-152	Proteobacteria	Rhizobacter	313	+
OTU-396	Proteobacteria	Rhodanobacte	451	+
OTU-1606	Proteobacteria	Rhodoplanes	609	+
OTU-126	Proteobacteria	Sphingomonas	2189	+
OTU-35	Proteobacteria	Unassigned	3541	+
OTU-39	Proteobacteria	Unassigned	2593	+
OTU-12	Proteobacteria	Unassigned	2121	+
OTU-199	Proteobacteria	Unassigned	1724	+
OTU-301	Proteobacteria	Unassigned	1159	+
OTU-60232	Proteobacteria	Unassigned	503	+
OTU-345	Proteobacteria	Unassigned	481	+
OTU-325	Proteobacteria	Unassigned	419	+
OTU-1216	Proteobacteria	Unassigned	363	+
OTU-27	Proteobacteria	Unassigned	340	+
OTU-3041	Proteobacteria	Unassigned	231	+
OTU-749	Proteobacteria	Unassigned	141	+
OTU-2854	Proteobacteria	Unassigned	102	+
OTU-193	Proteobacteria	uncultured	1496	+
OTU-183	Proteobacteria	uncultured	970	+

OTU-865	Proteobacteri	uncultured	702	+
OTU-935	Proteobacteri	uncultured	651	+
OTU-145	Proteobacteri	uncultured	605	+
OTU-63	Proteobacteri	uncultured	551	+
OTU-302	Proteobacteri	uncultured	324	+
OTU-5866	Proteobacteri	uncultured	246	+
OTU-269	Proteobacteri	uncultured ba	1148	+
OTU-184	Proteobacteri	uncultured ba	835	+
OTU-9433	Proteobacteri	uncultured ba	615	+
OTU-1127	Proteobacteri	uncultured ba	519	+
OTU-898	Proteobacteri	uncultured ba	461	+
OTU-7109	Proteobacteri	uncultured ba	438	+
OTU-409	Proteobacteri	uncultured ba	335	+
OTU-10553	Proteobacteri	uncultured ba	261	+
OTU-146969	Proteobacteri	uncultured ba	215	+
OTU-153	Proteobacteri	uncultured Co	1184	+
OTU-816	Proteobacteri	Variovorax	381	+
OTU-151	Verrucomicrol	Candidatus Uc	648	+
OTU-300	Verrucomicrol	Candidatus Uc	577	+
OTU-1373	Verrucomicrol	Candidatus Uc	384	+
OTU-29740	Verrucomicrol	Candidatus Uc	328	+
OTU-510	Verrucomicrol	Candidatus Uc	258	+
OTU-99	Verrucomicrol	Cephaloticocc	779	+
OTU-221	Verrucomicrol	Chthoniobacte	551	+
OTU-616	Verrucomicrol	Chthoniobacte	349	+
OTU-1581	Verrucomicrol	Chthoniobacte	297	+
OTU-1043	Verrucomicrol	Chthoniobacte	242	+
OTU-688	Verrucomicrol	Luteolibacter	320	+
OTU-445	Verrucomicrol	Roseimicrobiu	288	+
OTU-504	Verrucomicrol	Unassigned	841	+
OTU-377	Verrucomicrol	uncultured ba	1327	+
OTU-694	Verrucomicrol	uncultured ba	473	+

TABLE S2

OTU.ID	Phylum	Genus	CumAb
OTU-1	Acidobacteria	Bryobacter	4706
OTU-47	Acidobacteria	uncultured	3074
OTU-45	Acidobacteria	Unassigned	2611
OTU-24	Acidobacteria	RB41	2524
OTU-69	Acidobacteria	Subgroup 10	655
OTU-561	Acidobacteria	uncultured ba	495
OTU-352	Acidobacteria	uncultured Ac	493
OTU-154895	Acidobacteria	Unassigned	476
OTU-31773	Acidobacteria	Unassigned	454
OTU-555	Acidobacteria	Unassigned	442
OTU-4424	Acidobacteria	Candidatus Ko	397
OTU-7771	Acidobacteria	RB41	383
OTU-113001	Acidobacteria	Unassigned	350
OTU-4322	Acidobacteria	Unassigned	345
OTU-288	Acidobacteria	Paludibaculum	309
OTU-3623	Acidobacteria	Unassigned	297
OTU-152453	Acidobacteria	metagenome	238
OTU-735	Acidobacteria	Unassigned	212
OTU-121	Actinobacteria	Unassigned	653
OTU-264	Actinobacteria	Nocardioides	488
OTU-441	Actinobacteria	Nocardioides	327
OTU-4520	Actinobacteria	Oerskovia	304
OTU-388	Armatimonad	Chthonomona	340
OTU-105	Bacteroidetes	uncultured	1841
OTU-1372	Bacteroidetes	Flavisolibacter	695
OTU-187	Bacteroidetes	Ferruginibacte	691
OTU-502	Bacteroidetes	uncultured Ba	469
OTU-8461	Bacteroidetes	Ferruginibacte	469
OTU-2774	Bacteroidetes	uncultured ba	448
OTU-160	Bacteroidetes	Unassigned	418
OTU-2167	Bacteroidetes	uncultured	411
OTU-3288	Bacteroidetes	uncultured	404
OTU-466	Bacteroidetes	Flavisolibacter	303
OTU-71666	Bacteroidetes	Ferruginibacte	216
OTU-36	Bacteroidetes	uncultured	159
OTU-142	Chloroflexi	Anaerolinea	865
OTU-3090	Gemmatimon	uncultured	1392
OTU-1760	Gemmatimon	uncultured	1229
OTU-624	Gemmatimon	Gemmatimon	286
OTU-23	Proteobacteri	Lysobacter	5370
OTU-51	Proteobacteri	Unassigned	4654
OTU-15	Proteobacteri	Lysobacter	3113
OTU-62	Proteobacteri	Skermanella	2996
OTU-126	Proteobacteri	Sphingomonas	2563
OTU-199	Proteobacteri	Unassigned	1716
OTU-12	Proteobacteri	Unassigned	1696
OTU-92606	Proteobacteri	Sphingomonas	1557
OTU-132	Proteobacteri	Microvirga	1308

OTU-898	Proteobacteri	uncultured ba	465
OTU-197940	Proteobacteri	Unassigned	388
OTU-8525	Proteobacteri	Sphingomonas	342
OTU-3041	Proteobacteri	Unassigned	282
OTU-749	Proteobacteri	Unassigned	172
OTU-221	Verrucomicrol	Chthoniobacte	630
OTU-688	Verrucomicrol	Luteolibacter	450
