# Supplementary material for: Idiosyncratic invasion trajectories of human bacterial pathogens facing temperature disturbances in soil microbial communities
Source: Sci Rep. 2024 May 29;14:12375. doi: 10.1038/s41598-024-63284-5 (PMC11137084; doi:10.1038/s41598-024-63284-5)
Supplement: Supplementary file 2 — Supplementary Information. [file 41598_2024_63284_MOESM2_ESM.docx]

Table S1. List of OTUs with significant variation during invasion by *L. monocytogenes* L9.

Table S2. List of OTUs with significant variation during invasion by *K. pneumoniae* MGH 78578.
